# PINK1 attenuates mtDNA release in alveolar epithelial cells and TLR9 mediated profibrotic responses

**DOI:** 10.1371/journal.pone.0218003

**Published:** 2019-06-06

**Authors:** Marta Bueno, Daniel Zank, Ivette Buendia-Roldán, Kaitlin Fiedler, Brenton G. Mays, Diana Alvarez, John Sembrat, Brian Kimball, Jordan K. Bullock, James L. Martin, Mehdi Nouraie, Brett A. Kaufman, Mauricio Rojas, Annie Pardo, Moisés Selman, Ana L. Mora

**Affiliations:** 1 Division of Pulmonary Allergy and Critical Care Medicine, Department of Medicine, University of Pittsburgh, Pittsburgh, Pennsylvania, United States of America; 2 Aging Institute, Department of Medicine, University of Pittsburgh, Pittsburgh, Pennsylvania, United States of America; 3 Vascular Medicine Institute, Department of Medicine, University of Pittsburgh, Pittsburgh, Pennsylvania, United States of America; 4 Instituto Nacional de Enfermedades Respiratorias “Ismael Cosio Villegas”, Mexico City, Distrito Federal, México; 5 The Dorothy P. and Richard P. Simmons Center for Interstitial Lung Diseases, University of Pittsburgh, Pittsburgh, Pennsylvania, United States of America; 6 Center for Metabolism and Mitochondrial Medicine, Division of Cardiology, Department of Medicine, University of Pittsburgh, Pittsburgh, Pennsylvania, United States of America; 7 McGowan Institute of Regenerative Medicine, University of Pittsburgh, Pittsburgh, Pennsylvania, United States of America; 8 Facultad de Ciencias, Universidad Nacional Autónoma de México, Mexico City, Distrito Federal, México; National Institutes of Health, UNITED STATES

## Abstract

We have previously shown that endoplasmic reticulum stress (ER stress) represses the PTEN inducible kinase 1 (PINK1) in lung type II alveolar epithelial cells (AECII) reducing mitophagy and increasing the susceptibility to lung fibrosis. Although increased circulating mitochondrial DNA (mtDNA) has been reported in chronic lung diseases, the contribution of mitophagy in the modulation of mitochondrial DAMP release and activation of profibrotic responses is unknown. In this study, we show that ER stress and PINK1 deficiency in AECII led to mitochondrial stress with significant oxidation and damage of mtDNA and subsequent extracellular release. Extracellular mtDNA was recognized by TLR9 in AECII by an endocytic-dependent pathway. PINK1 deficiency-dependent mtDNA release promoted activation of TLR9 and triggered secretion of the profibrotic factor TGF-β which was rescued by PINK1 overexpression. Enhanced mtDNA oxidation and damage were found in aging and IPF human lungs and, in concordance, levels of circulating mtDNA were significantly elevated in plasma and bronchoalveolar lavage (BAL) from patients with IPF. Free mtDNA was found elevated in other ILDs with low expression of PINK1 including hypersensitivity pneumonitis and autoimmune interstitial lung diseases. These results support a role for PINK1 mediated mitophagy in the attenuation of mitochondrial damage associated molecular patterns (DAMP) release and control of TGF-β mediated profibrotic responses.

## Introduction

Idiopathic pulmonary fibrosis (IPF) is a progressive and incurable interstitial lung disease of unknown etiology with median survival of only 2 to 3 years from the time of diagnosis [1]. In the pathogenesis of IPF, chronic microinjury and aberrant activation of type II (AECII) epithelial cells drive accumulation of fibroblasts and myofibroblasts with exuberant deposition of extracellular matrix and abnormal remodeling [2, 3]. Our previous work has shown that AECII in IPF lungs contain swollen and dysfunctional mitochondria secondary to deficiency of a key mediator of mitochondrial homeostasis and mitophagy, the PTEN-induced putative kinase 1 (PINK1) [4, 5].

Owing to its prokaryotic origins, mitochondrial DNA (mtDNA) contains unmethylated CpG islands akin to bacterial DNA [6], which are recognized by several innate immune pattern recognition receptors including the NLRP3 inflammasome system, Toll-like receptor 9 (TLR9), and the cyclic GMP-AMP synthase-stimulator of interferon genes (cGAS-STING) pathway [7–10]. In addition, activation of TLR9 has been shown to be integral to the development of vascular dysfunction in hypertension, heart failure secondary to myocarditis and liver fibrosis in animal models [11–13] suggesting that TLR9 signaling plays a role in mediating pathologic responses to released mtDNA as a danger-associated molecular patterns (DAMPs). Increased levels of circulating mtDNA are associated with tissue injury and disease [14–17], suggesting that mitochondrial DAMPs play a role in the development or severity of human disease [14]. Recently, circulating mtDNA has been proposed as a predictor of mortality in IPF [18].

We hypothesize that mitochondrial dysfunction in AECIIs increase the release of mtDNA that can function as a DAMP and autonomously activate TLR9 to initiate downstream inflammatory and pro-fibrotic signaling.

## Materials and methods

### Study approval

Human lung tissue used for mitochondria isolation was collected from excess pathologic tissue after lung transplantation and organ donation, under University of Pittsburgh IRB‐ and CORID‐approved protocols (970946, PRO14010265, and CORID No. 300). Samples from Mexican patients were acquired at the Interstitial Lung Disease Clinic of the National Institute of Respiratory Diseases in Mexico City. Plasma and BAL samples were obtained under protocols approved by the local ethics committees, and all participants gave written informed consent.

All animal protocols were approved by the IACUC of the University of Pittsburgh (protocol number 16119337) and adhered to NIH guidelines for the use of experimental animals.

### Human studies

Demographic characteristics of the Pittsburgh cohort used for mitochondria isolation or total lung lysates are described in S1–S4 Tables. Demographic characteristics of the Mexican cohort (plasma and BAL) are reported in S5 Table.

### Animal studies

PINK1-deficient mice were bred in house (maintained as a heterozygous line) to generate PINK1 WT (+/+) and PINK1 KO (–/–) littermates [4]. PINK1 mice were sacrificed at different ages (3-, 8- and 12-month old) for organ harvest to isolate mitochondria and mtDNA. BAL from 6 months old PINK1 mice was collected by using two consecutive instillations of PBS (0.5 ml) at room temperature and was centrifuged at 1500 rpm at 4°C for 5 min; supernatants were collected and stored at −80°C for mtDNA measurement. C57BL6 mice were acquired from Jackson Labs (Bar Harbor, ME) and TLR9 KO mice were obtained from the Shlomchik Lab (University of Pittsburgh) [19]. Six-month-old mice (C56BL6 and TLR9 KO) were used to procure the lung tissue for precision-cut lung slices. Mice were euthanized by isofluorane overdose followed by cervical dislocation according to the guidelines from the Institutional Animal Care and Use Committee from the University of Pittsburgh.

### Cell lines and maintenance

Human lung adenocarcinoma cells (cell line A549, ATCC CCL‐185, passages 6 to 8) were cultured in DMEM (Gibco) with 10% FBS (Gibco) and 50 U/ml penicillin with 50 μg/ml streptomycin (Gibco). Mouse lung epithelial cells (cell line MLE12, ATCC CRL-2110, passages 5 to 6) were maintained in HITES medium (Dulbecco’s medium: Ham’s F12, 50:50 mix; insulin 0.005mg/ml; transferrin 0.01 mg/ml; sodium selenite 30nM; hydrocortisone 10nM; β-estradiol 10nM; HEPES 10nM, L-glutamine 10mM) supplemented with 2% FBS (Gibco) and 50 U/ml penicillin with 50 μg/ml streptomycin (Gibco). Primary human pulmonary alveolar epithelial cells (AECIIs, ScienCell #3200, cryopreserved at passage 0) grew overnight in complete alveolar epithelial cell medium (ScienCell #3201) over a poly‐L‐lysine coated plate (ScienCell #0403) before been treated. Primary normal and IPF fibroblast were isolated as previously described [20], cultured in F12 nutrient Ham Medium (Gibco) with 10% FBS (Gibco) and 1% of antimycotic-antibiotic (Gibco) and used in passages 5 to 9. Cells were cultured at 37°C, in 5% CO_2_.

### DNA isolation for quantification and stimulation

Mitochondrial DNA was detected and quantified as previously described [16]. In detail, a volume of BAL fluid (100 μl), plasma (100 μl) or cell supernatant (500 μl) was further filtrated with a 0.2 μm PES membrane (Sartorius VS0171). DNA was isolated from the ultra-filtrated fluid using the QiAmp DNA extraction kit (Qiagen, 51306) following manufacturer’s recommendations. Mitochondrial DNA present in the samples was assessed by qPCR using ND1 and mt16S in human samples, while ND2 was used for the murine derived samples. Nuclear DNA was amplify using B2M for human or GAPDH for mouse. Primer sequences were as follows: Gapdh forward, CCTGCACCACCAACTGCTTAG; Gapdh reverse, GTGGATGCAGGGATGATGTTC; Nd2 forward, CCCATTCCACTTCTGATTACC; Nd2 reverse, ATGATAGTAGAGTTGAGTAGCG; B2M forward, TGCTGTCTCCATGTTTGATGTATCT; B2M reverse, TCTCTGCTCCCCACCTCTAGGT; mt16S forward, GCCTTCCCCCGTAAATGATA, mt16S reverse, TTATGCGATTACCGGGCTCT; Nd1 forward: CCCTAAAACCCGCCACATCT; Nd1 reverse: GAGCGATGGTGAGAGCTAAGGT. To generate the mtDNA standard curve for mtDNA copy quantification (copies per μl), a PCR fragment of around 3Kb was cloned from human or murine isolated mtDNA containing the sequences of interest ND1 and mt16S for human (Human NC_0122920, fragment 2699–5272 bp) and Nd2 for mouse (Mouse NC-005089, fragment 1533–4648 bp).

For mtDNA or gDNA stimulation, intact mitochondria and nuclei were isolated from fresh lung tissue or cell culture by differential centrifugation in STE buffer (250 mM sucrose, 10 mM Tris, 1 mM EGTA, pH 7.4) as previously described [4]. DNA was isolated from mitochondria (mtDNA) or nuclei (gDNA) using QiAmp DNA extraction kit (Qiagen, 51306), quantify spectroscopically and stored at -80C in “one-use only” aliquots. To assess the purity of the mtDNA isolation for stimulation, human or mouse derived mtDNA was cut at a single site with BglII or PvuII (respectively) and run in a 0.5% agarose gel to generate a sharp band at 16.5kb.

### Mitochondrial DNA oxidation and lesion quantification

Mitochondrial DNA oxidation was evaluated by ELISA detection of 8-hydroxy-2’-deoxyguanosine (Cayman #589320) according to the manufacturer’s protocol in 3 μg of isolated mtDNA from freshly isolated total lung tissue mitochondria. Mitochondria DNA lesions were quantified using long-run qPCR technique for DNA-damage quantification (LORD-Q) method calculating 10kb lesions using amplification efficiency from standard curve based on Ct values [21, 22].

### Experimental cell culture, gene silencing and treatments

To knock down protein expression, A549 cells were transfected with siRNA for PINK1 (s35168, Thermo Fisher), TLR9 (s288772, Thermo Fisher) or scramble siRNA controls (Select Negative Control No. 2 siRNA, Thermo Fisher 4390847). Treatments were performed beginning 24 hr after transfection and for an additional 24 hr.

Supernatant and cell pellets here harvested at different time point from cells treated with tunicamycin (TM; Sigma‐Aldrich) (AEC: 0.1 μg/ml; A549: 1 μg/ml) or DMSO (Sigma‐Aldrich; AEC: 0.002%; A549: 0.02%) as vehicle control.

Cells were transfected with PINK1-3xFlag in pReceiver M14 (Genecopoeia) or GFP pReceiver M14 (control) for PINK1 overexpression using a transfecting agent (Lipofectamine 3000, Thermo Fisher). All the transfections were performed in no antibiotic and low FBS (1%) media for 6h, then the media was change to DMEM (Gibco) with 10% FBS (Gibco) and 50 U/ml penicillin with 50 μg/ml streptomycin (Gibco) for the rest of the experimental set-up. Treatments with TM were performed 24h after transfection, and supernatant was harvested 24h post stilulation.

Supernatant and cell pellets (from A549 and primary human alveolar epithelial cells) here harvested at 24h after stimulation with extracellular mtDNA or gDNA (1μg/ml) in the presence of DNAse (1U/ml). Pre-treatment with TLR9 agonist ODN M362 (5’- TCG TCG TCG TTC GAA CGA CGT TGA T-3’ phosphorothioated bases, IDT; 1μM), TLR9 antagonist ODN TAG (5’- TTT AGG GTT AGG GTT AGG GTT AGG G -3’ phosphorothioated bases, IDT; 1μM), NFκB inhibitor BAY11-7082 (5μM; Calbiochem) dynasore (80μM; Sigma) or chloroquine (50μM; Sigma) was performed 1h prior to exogenous DNA stimulation. Internal stimulation with mtDNA or gDNA (1μg/ml) was performed using a transfecting agent (Lipofectamine 3000, Thermo Fisher).

Supernatant and cell pellets (from human primary fibroblast derived from IPF patients or aged-matched controls) here harvested after 48h of culture in the presence and absence of stimulation with 1μg/ml of exogenous mtDNA or TGF-β (5ng/ml).

For the co-culture experiments, A549 cells were grown in transwell inserts overnight and then stimulated with PBS 1μg/ml mtDNA or tunicamycin 10μg/ml for 2h. Supernatant was collected to confirm increase in TGF-β release and then epithelial cells were washed extensively with cell media. Those A549 cells in transwell inserts were place over overnight cultures of human primary fibroblast derived from IPF patients or aged-matched controls. After 48h of co-culture, cell pellets from epithelial cells and fibroblast were collected for RNA extraction.

### RNA isolation and qRT-PCR

Purification of RNA was performed in cells and lung tissue using RNA isolation kits (Qiagen 74104), according to the manufacturer’s recommendations. To quantify relative gene expression, 1-step qRT-PCR (Affymetrix 75700) was performed for the genes of interest and normalized using 18S RNA as housekeeping gene. Premixed primers and probes were used for TGFB1, TLR9, PINK1, COL1A1, FN1, ACTA2, NOX4, IL6 and IL1B (Integrated DNA Technologies, assay numbers in S6 Table).

### Transforming Growth Factor beta 1 quantification

Levels of total TGF-β1 in BAL or cell culture supernatant were quantified using ELISA kits (ThermoFisher Scientific 88-850-22) following the manufacturer’s protocol.

### Precision-cut lung slices (PCLS)

For human PCLS, single lung segments were dissected and warmed in a 37°C water bath for 30 min. Low-melting-point agarose (2%, Invitrogen Ultrapure) in sterile medium (DMEM; Gibco, supplemented with 50 U/ml penicillin with 50 μg/ml streptomycin and 2.5 μg/ml amphotericin B) was maintained at 37°C. The lung segments were filled with agarose (by instillation into airways with an 18-gauge cannula) and inspected for appropriate expansion, followed by airway clamping and packing in ice for 30 min (or until the agarose had set). Tissue was cut to the required block size (2 cm x 1cm x 1 cm) and sliced in ice-cold saline with a vibratome (28mm/sec; Leica VT 1200) at a slice thickness of 300 mm. Uniform slices (1 cm x 1 cm) were cultured in 0.5ml of DMEM containing penicillin-streptomycin and amphotericin B without serum in 24-well dishes at 37°C in a tissue incubator with 5% CO_2_ for 2h. Then, the slices were cultured in DMEM (Gibco) with 10% FBS (Gibco) and 50 U/ml penicillin with 50 μg/ml streptomycin (Gibco) overnight. Finally, slices were stimulated with different doses of exogenous human mtDNA. Supernatant and slice were harvested 24h after stimulation for analysis.

For murine derived PCLS, wild type or knock-out mice were anaesthetised with a mixture of ketamine and xylazine hydrochloride. After intubation and dissection of the diaphragm, lungs were loaded with low-melting-point agarose (2%) in sterile medium (DMEM; Gibco, supplemented with 50 U/ml penicillin with 50 μg/ml streptomycin and 2.5 μg/ml amphotericin B) at 37°C. The trachea was ligated with thread to retain the agarose inside the lung. The lung was excised, transferred into a tube with medium and cooled on ice for 10 min to allow the agarose hard set. The lobes were separated and cut with a vibratome (28mm/sec; Leica VT 1200) at a slice thickness of 300 mm. Uniform slices were sectioned into 1 cm x 1 cm sections and cultured in 0.5ml of DMEM containing penicillin-streptomycin and amphotericin B without serum in 24-well dishes at 37°C in a tissue incubator with 5% CO_2_. The medium was changed after 2h to DMEM (Gibco) with 10% FBS (Gibco) and 50 U/ml penicillin with 50 μg/ml streptomycin (Gibco) and kept in culture overnight. Slices were exposed to 1μg/ml mtDNA from PINK1+/+ mice (WT) or PINK1 -/- (KO) mice and then supernatant and slice were harvested for analysis after 24h.

For viability assessment, (live/dead) extra slices from the same agarose block were taken (at time of preparation) and stained with AOPI Staining Solution (Fisher, NC0285242).

### Statistical analysis

Differences between groups were calculated by 2-tailed unpaired Student’s t test with Welch’s correction or by non-parametric one- or two-way ANOVA followed by post-hoc tests (Sidak’s multiple comparisons test). Correlations were calculated by the Spearman method. Statistical significance was considered if the p < 0.05. Statistical analyses were carried out using Prism 7 (GraphPad) and Stata 14.2 (StataCorp) software.

## Results

### PINK1 deficiency lead to selective cellular release of mitochondrial DNA

**Fig 1:**
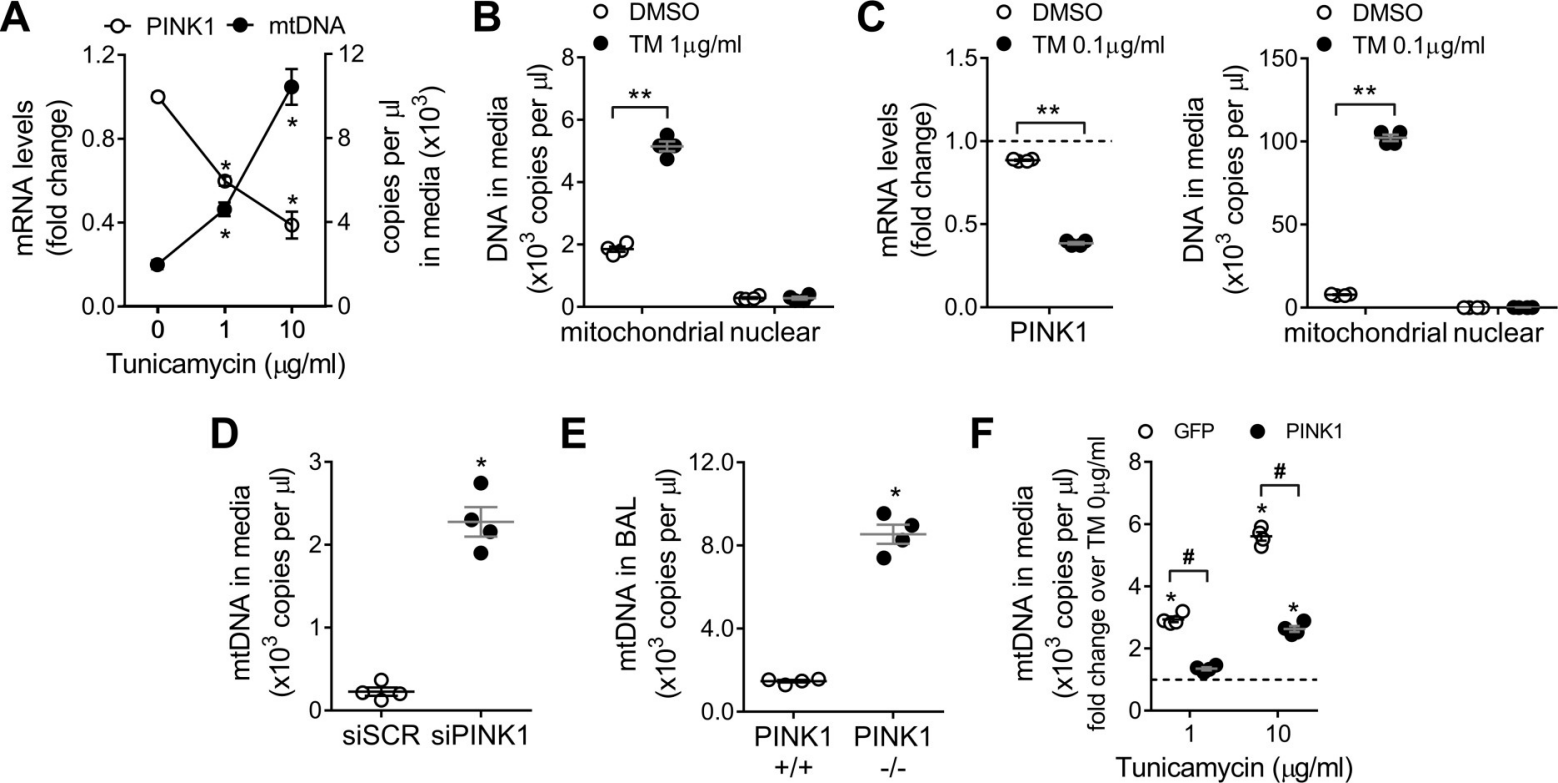
PINK1 deficient lung epithelial cells selectively release mitochondrial DNA. (A) PINK1 mRNA levels and mtDNA copies in culture media in A549 cells treated with increasing doses of TM for 24h (n = 3; *p<0.01 vs TM 0μg/ml; two-way ANOVA with multiple comparison). (B) Mitochondrial and nuclear DNA copies detected in culture media in A549 cells treated with TM 1μg/ml for 24h (n = 4; **p<0.0001; two-way ANOVA with multiple comparison). (C) PINK1 mRNA levels in primary human alveolar epithelial cells treated 24h with TM 0.1μg/ml (n = 4; **p<0.0001; unpaired t-test). Mitochondrial and nuclear DNA copies detected in culture media in primary human alveolar epithelial cells treated 24h with TM 0.1μg/ml. (n = 4; **p<0.0001; two-way ANOVA with multiple comparison). (D) MtDNA copies in culture media in PINK1 knock-down A549 cells (n = 3, *p<0.001 vs scramble; unpaired t-test). (E) MtDNA copies detected in the BAL fluid of PINK1 +/+ and -/- mice (n = 4, *p<0.001 vs PINK1 +/+; unpaired t-test). (F) MtDNA copies in culture media in A549 cells overexpressing GFP or PINK1 then treated with increasing doses of TM for 24h (n = 4; *p<0.01 vs TM 0μg/ml, #p<0.001 vs GFP; two-way ANOVA with multiple comparison). Dot plots represent mean ± SEM. See S1 Fig for details about ER stress markers levels after TM treatment and knock-down / overexpression efficiencies.

We have previously shown that IPF lung epithelial cells present low levels of PINK1 [4], driven by persistent ER stress [23] and chronic transcriptional repression [24], that dysregulate mitochondria homeostasis and induce accumulation of damaged mitochondria. PINK1 deficiency is linked to increase opening of the mitochondrial permeability transition pore (mPTP), which allows nonselective traffic to the cytosol liberating mitochondrial content, including mtDNA [25]. To investigate the relationship between ER stress, PINK1 deficiency and mtDNA release, A549 cells were treated for 24h with increasing concentrations of tunicamycin (TM) to induce ER stress (S1A Fig). PINK1 transcript was reduced after ER stress stimulation. Concomitantly, mtDNA was detected in the cell culture supernatant in a dose dependent manner (Fig 1A). In high contrast, levels of extracellular nuclear DNA in the cell media were very low and were not different in the presence or absence of ER-stress induced PINK1 repression (Fig 1B). Primary human alveolar epithelial cells show higher number of mtDNA copies released (Fig 1C) when subjected to ER stress (S1B Fig), even at low concentrations of tunicamycin (0.1 μg/ml) with no differential detection of nuclear DNA in the supernatant (Fig 1C). Similarly, knockdown of PINK1 expression in A549 cells (S1C Fig) resulted in increased release of mtDNA (Fig 1D). To determine if selective release of mtDNA linked to PINK1 deficiency occurs *in vivo*, we analyzed levels of mtDNA in BAL fluid of wild type and PINK1 knockout mice (S1D Fig) and found a significant increase in mtDNA in the PINK1 -/- mice (Fig 1E). This ER stress induced mtDNA release is driven by the loss of PINK1 (Fig 1F), since the overexpression of PINK1 (S1E Fig) in TM-treated cells inhibits mtDNA leak to the cell media (Fig 1F). These data show that, *in vitro* and *in vivo*, PINK1 deficient lung alveolar epithelial cells (induced by ER stress or by silencing/knockout) release extracellular mtDNA.

### PINK1 deficiency and isolated mtDNA increase pro-inflammatory and pro-fibrotic cytokines release

**Fig 2:**
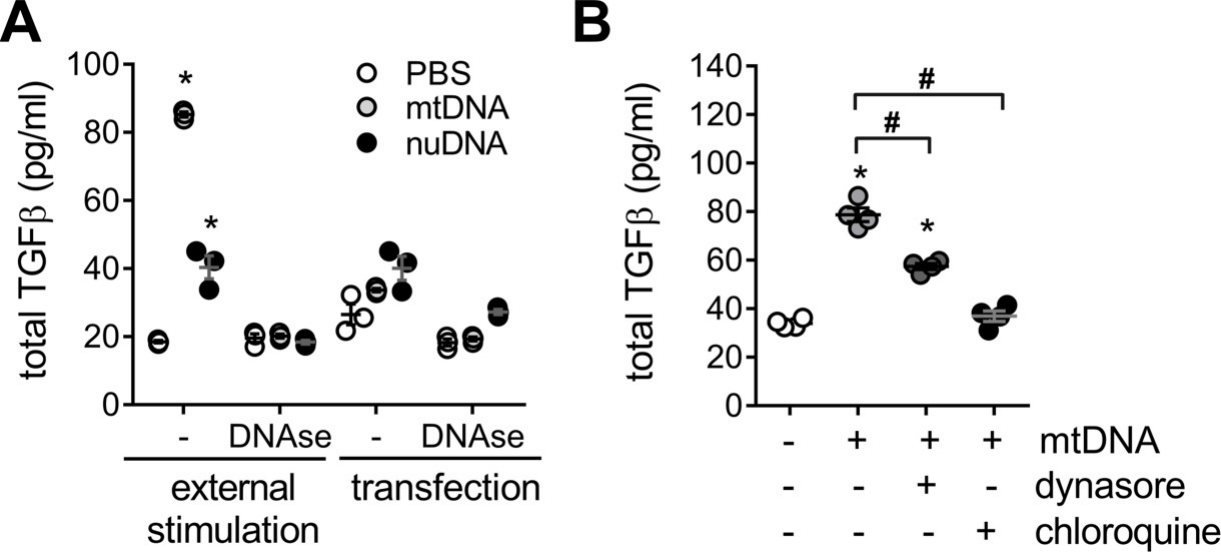
Cells stimulated with free mitochondrial DNA increase TGF-β release. (A) TGF-β in cell media from A549 treated with extracellular mtDNA or nuclear nDNA (1μg/ml, 24h) and it is absent in the presence of DNAse (1U/ml), using PBS as vehicle (n = 3; *p<0.01 vs PBS; two-way ANOVA with multiple comparison). (B) TFG-β release induced by exogenous mtDNA treatment (1μg/ml, 24h) in A549 cells pretreated for 1h, prior stimulation, with endocytosis blockers (dynasore 80μM or chloroquine 50μM) (n = 4; *p<0.01 vs PBS, ^#^p<0.01; one-way ANOVA with multiple comparison). Dot plots represent mean ± SEM.

To confirm that mtDNA specifically triggered pro-inflammatory and pro-fibrotic responses in lung epithelial cells, we analyzed A549 cells stimulated with exogenous mtDNA (1μg/ml) in the media. TGF-β release was increased by mtDNA stimulation (Fig 2A). In addition, TGF-β production was mtDNA concentration-dependent also in mouse lung epithelial cell lines as MLE12 (S2A Fig). As pro-inflammatory marker, we evaluated IL-6 expression that was upregulated after mtDNA stimulation (S2B Fig). IL-6 mRNA transcript level was significantly higher in whole lung lysates of PINK1 knockout mice, while total TGF-β was 10-fold higher in the BAL fluid of the deficient mice (S2C Fig). Similarly, IL-6 and TGF-β increased in A549 cells with PINK1 siRNA knockdown for 48 hours (S2D Fig). This TGF-β response was specific to mtDNA external stimulation since pre-treatment of media with DNAse (1U/ml) abrogated this response, and the stimulation with nuclear DNA (1μg/ml) had a much lesser effect than mtDNA (Fig 2A). Additionally, we tested transfection of mtDNA or nuclear DNA into A549 cells, but this method did not significantly change the expression of TGF-β (Fig 2A). To test if the trigger of this TGF-β activation resides in the cell surface, cells were concurrently stimulated with mtDNA and an endosome active agent (Fig 2B). Blocking early endocytosis with Dynasore or later endosomal acidification with chloroquine decrease the release of TGF-β to the media after mtDNA stimulation (Fig 2B). Altogether, this data suggest that mtDNA can externally trigger pro-inflammatory and pro-fibrotic responses in lung epithelial cells. It also suggests that the stimulus driving the activation of TGF-β occurs on the cell surface and on the inner surface of the endosome after endocytic uptake of mtDNA rather than in the cytosol.

### Toll-like receptor 9 mediates TGF-β release in response to mitochondrial DNA

**Fig 3:**
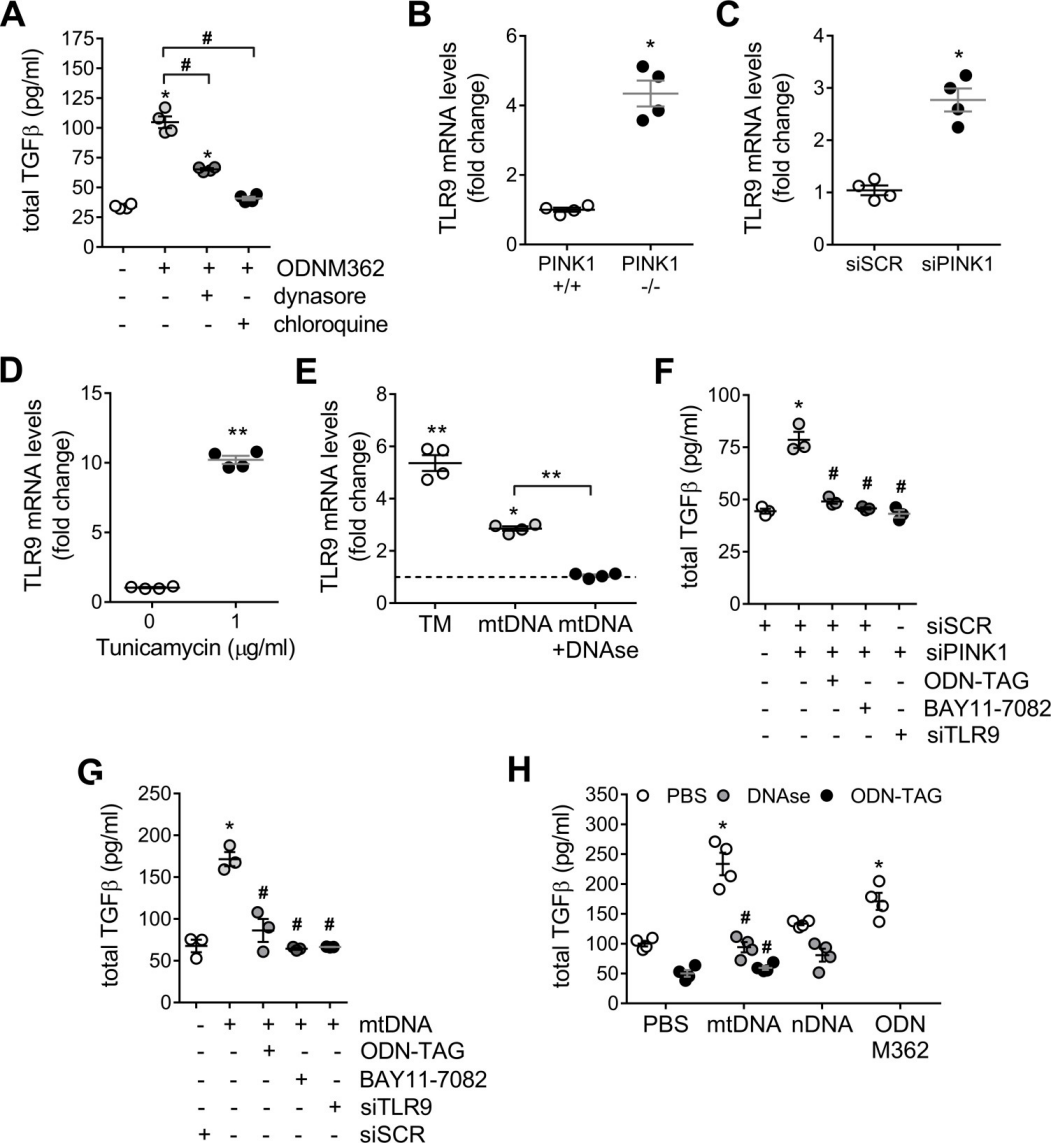
TLR9 mediates TGF-β release in response to mitochondrial DNA. (A) Pre-treatment with endocytosis blockers (dynasore 80μM or chloroquine 50μM) for 1h reduced TFG-β released induced by TRL9 agonist ODN M362 (2μM) after 24h (n = 4; *p<0.01 vs PBS, ^#^p<0.01; one-way ANOVA with multiple comparison). TLR9 mRNA transcript levels in total lung lysate from PINK1 -/- mice (B), A549 cells treated with PINK1 siRNA for 48h (C) or A549 cells treated with 1μg/ml of tunicamycin for 24h (D) (n = 4; *p<0.05, **p<0.01; unpaired t-test). (E) TLR9 mRNA transcript levels in primary human lung epithelial cells (E) after 24 of 0.1μg/ml of tunicamycin or 1 μg/mL exogenous mtDNA in the presence or absence of DNAse (1U/ml) (n = 4; *p<0.05 vs unstimulated, **p<0.01; one-way ANOVA with multiple comparison). TGF-β release in A549 cells were treated with PINK1 siRNA (F) or stimulated with 1 μg/mL exogenous mtDNA for 24h (G) with pre-treatment with a TLR9 antagonist (ODN-TAG, 1μM), the NFκB antagonist BAY11-7082 (5μM), or by silencing TLR9 48h prior stimulation (n = 3; *p<0.001 vs PBS, #p<0.001 vs siPINK1 or mtDNA; one-way ANOVA with multiple comparison). (H) TGF-β release in primary human lung epithelial cells after stimulations with extracellular mtDNA (1μg/ml) in the presence of DNAse (1U/ml), pre-treatments with TRL9 antagonist ODN-TAG (1μM) or exposed to 1μg/ml of nDNA after 24h. TRL9 agonist ODN M362 (1μM) (n = 4; *p<0.001 vs PBS, #p<0.001 vs mtDNA; two-way ANOVA with multiple comparison). Dot plots represent mean ± SEM. See S1C and S1D Fig and S3A Fig for knock-down efficiencies.

Non-mitochondrial located free mtDNA can engage multiple innate immunity pattern recognition receptors. Since the TGF-β response to mtDNA stimulation is mediated by endocytosis (Fig 2B) and TLR9 is known to translocate to the endosome, we examined the role of TLR9 in upregulating pro-inflammatory and pro-fibrotic cytokines in the setting of PINK1 deficiency and mtDNA stimulation. Cells stimulated with a TLR9 agonist (ODN M362) upregulated the secretion of TGF-β to the culture media, however in the presence of dynasore or chloroquine, this upregulation was reduced (Fig 3A). As well, we examined the effect of PINK1 deficiency on TLR9 expression. We found that TLR9 expression was upregulated in total lung lysate of PINK1 knockout mice when compared their wild type littermates (Fig 3B), at 48 hours after PINK1 siRNA knockdown in A549 cells (Fig 3C) as well as after treatment with tunicamycin (Fig 3D). In primary human lung epithelial cells, TRL9 expression is upregulated not only when PINK1 is reduced by tunicamycin treatment but also in the presence of mtDNA external stimulation (Fig 3E). Furthermore, when primary human lung epithelial cells are pretreated with DNAse and then stimulated with mtDNA, TLR9 expression upregulation is eliminated (Fig 3E).

In addition, TLR9 signaling can induce pro-inflammatory and pro-fibrotic signaling through the NFκB pathway [26]. To demonstrate the involvement of the TLR9- NFκB signaling pathway, A549 cells were treated with PINK1 siRNA to induce mtDNA release (Fig 3F) or were directly stimulated with 1 μg/mL extracellular mtDNA (Fig 3G). Both cases resulted in a statistically significant increase in TGF-β release, and, concurrent pre-treatment with a TLR9 antagonist (ODN-TAG), the NFκB antagonist BAY11-7082, or transfection with TLR9 siRNA returned the level of TGF-β to baseline (Fig 3F and 3G). Finally, this TLR9-mediated response in primary human lung epithelial is more sensitive to mtDNA stimulation, since addition of nuclear DNA only induced a very modest increase in TGF-β release and treatment with mtDNA had a similar effect when compared to treatment with the TLR9 agonist, ODN M362 (Fig 3H). Concurrent treatment of primary human lung epithelial cells with mtDNA and either DNAse or a TLR9 antagonist resulted in similar mitigation of induced TGF-β (Fig 3H). Similarly, IL-6 mRNA transcripts increased to comparable levels when primary human lung epithelial cells were treated with mtDNA or with ODN M362. Pretreating with DNAse or ODN M362, once again, returned TGF-β and IL-6 expression to baseline (S3B Fig). In sum, these data suggest that, in lung epithelial cells, extracellular mtDNA increases TGF-β via the TRL9-NFκB signaling pathway and indicates the presence of a positive feedback loop with activation of TLR9 expression upregulation.

### IPF and PINK1-deficient lungs have increased oxidation and instability of mitochondrial DNA

**Fig 4:**
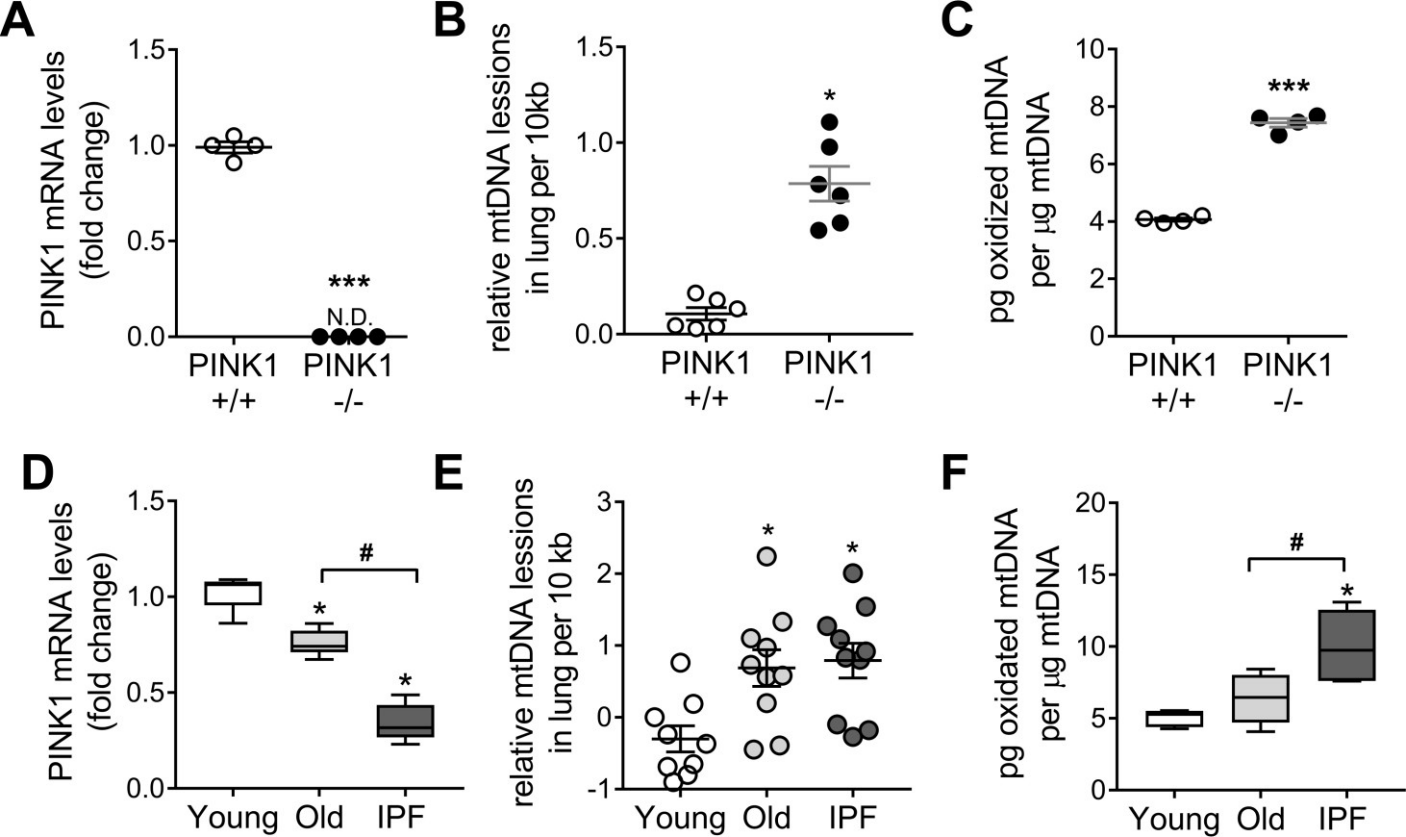
IPF and PINK1 deficient lungs show higher mitochondrial DNA oxidation. (A) PINK1 transcript levels in total lung of PINK1 deficient mice (n = 4; ***p<0.001, N.D non-detected; unpaired t-test). (B) Mitochondrial DNA lesions, by LORD-Q qPCR, in PINK WT and KO mice (n = 6; *p<0.01; unpaired t-test). (C) Oxidative damage in lung mtDNA (by 8-OH-dG per μg of analyzed mtDNA) of PINK1 KO mice (n = 4; ***p<0.001; unpaired t-test). See S4A Fig for aged mice data. (D) Transcript levels of PINK1 in young and old donor lung tissue and in IPF patients. (Min-to-max with median; n = 10; *p<0.01 vs young, ^#^p<0.01; one-way ANOVA with multiple comparison). (E) Mitochondrial DNA lesion by LORD-Q qPCR in lungs of young, old and IPF patients (n = 10; *p<0.01 vs young; one-way ANOVA with multiple comparison). (F) Oxidative damage in lung mtDNA (by 8-OH-dG per μg of analyzed mtDNA) of IPF patients (Min-to-max with median; n = 6; *p<0.01 vs young, ^#^p<0.01; one-way ANOVA with multiple comparison). Dot plots represent mean ± SEM. See S1–S3 Tables for demographics of panel D-F.

Since PINK1 has a role in mtDNA metabolism [4, 27], we analyzed mtDNA damage in lungs of PINK1 deficient mice (Fig 4A). We quantified DNA lesions in lung mtDNA using a long extension qPCR technique for DNA-damage quantification (LORD-Q) method [21, 22] and show more lesion in the PINK1 -/- mice (Fig 4B). To determine whether oxidation of mtDNA was increased and/or accumulative, we analyzed the content of 8-hydroxy-2'-deoxyguanosine (8-OH-dG), the most frequent oxidative DNA modification, on purified mtDNA samples from PINK1 WT and KO mice at different ages. At 3 months of age, mtDNA oxidation was greater in PINK1 KO mice (Fig 4C) and increases with age, in both WT and KO mice (S4A Fig). To determine if changes in PINK1 expression can influence the extent of mtDNA lesions caused by oxidative stress, we exposed A549 lung epithelial cells (with and without silencing PINK1 expression) to 400 μM H_2_O_2_ for 15 minutes. The mtDNA of PINK1 depleted cells (S4B Fig) were more susceptible to genotoxic stimulation relative to a scrambled control. In addition, after washing the cells for 1h, siPINK1 cells were unable to repair its damaged mtDNA as shown by the persistence of lesions (S4C Fig). In humans, as the lung age, the levels of PINK1 drop and further decrease in the IPF lung [4] (Fig 4D and S1 Table). Mitochondrial DNA lesions were higher in old and IPF lungs compared to the young group (Fig 4E and S2 Table). These data show that the lung accumulate unrepaired mtDNA damage with aging and disease. Increased 8-OH-dG content (per μg of isolated mtDNA) was found in lungs from IPF patients and age-matched donor control when compared with young donor lungs (Fig 4F and S3 Table). Altogether, these data confirm that PINK1 deficiency, in the PINK1 KO mice or in aged and IPF lungs [4], accumulates mtDNA damage consistent with unrepaired oxidative DNA damage and senescence [24].

### Release of oxidized mitochondrial DNA increases downstream production of TGF-β

**Fig 5:**
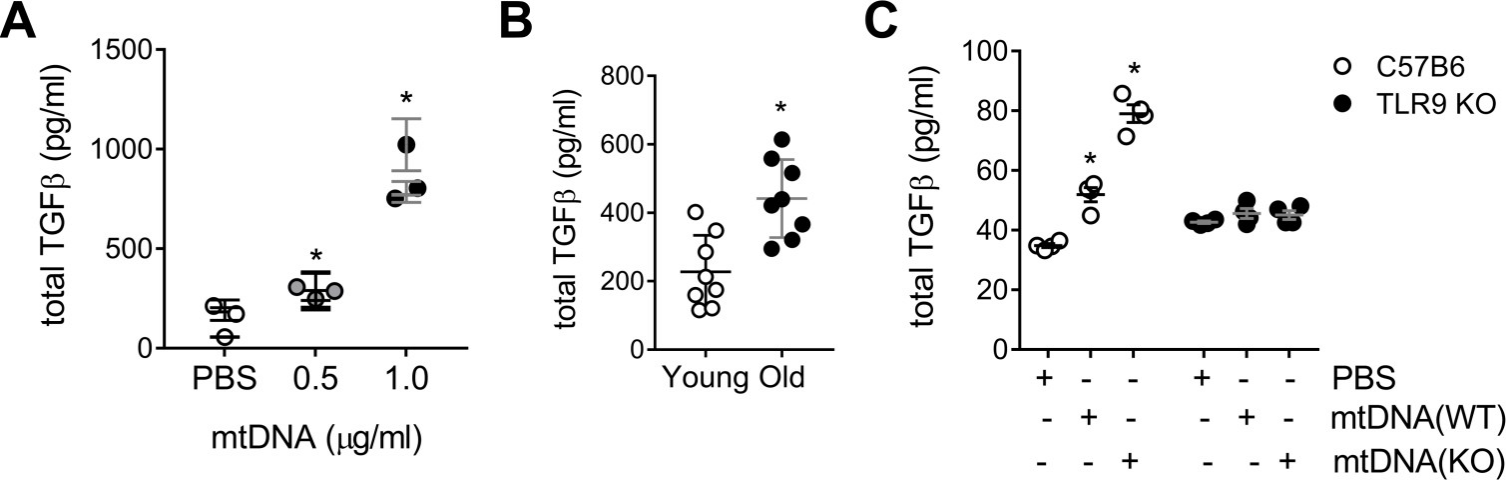
Oxidized mtDNA increases downstream production of TGF-β. (A) TFG-β release in precision-cut lung slices (PCLS) from healthy human donors (60±3) treated with different doses of exogenous mtDNA after 24h (n = 3 patients, n = 6 per patient; *p<0.001 vs PBS; one-way ANOVA with multiple comparison). (B) TFG-β release in human PLCS after 24h of stimulation with 1μg/ml mtDNA isolated from young (35yo) or old (87yo) donor lungs (n = 8 per patient; *p<0.001; unpaired t-test). (C) TGF-β (24h) in C57BL6 or TLR9 mouse PCLS after stimulation with 1μg/ml mtDNA from PINK1+/+ mice (WT) or PINK1 -/- (KO) mice. (n = 4; *p<0.001 vs PBS; two-way ANOVA with multiple comparison). Dot plots represent mean ± SEM. See S5 Fig for representative PCLS viability staining (S5A Fig for human PCLS, S5B Fig for mouse PCLS).

To confirm that mtDNA exposure induced a pro-fibrotic response in human lung, precision-cut lung slices (PCLS) of control donors (60±3) were treated with increasing doses of mtDNA for 24 hours (S5A Fig). We observed a dose-dependent increase in the release of total TGF-β upon stimulation (Fig 5A). In addition, higher TGF-β release happened when the mtDNA was isolated from lung of older patients (87yo) vs younger counterparts (with higher expression of PINK1); suggesting a role for the mtDNA oxidation state in the induction of the pro-fibrotic response (Fig 5B). To evaluate further the role of mtDNA oxidation state upon PINK1 deficiency, we cultured murine PCLS derived from C57BL6 mice and treated them with mtDNA isolated from PINK1 WT or KO mice (S5B Fig). A more oxidized mtDNA from PINK1 KO mice induced a higher upregulation of TGF-β secretion when compared to PINK1 WT derived mtDNA (Fig 5C). To confirm the role of the TLR9 signaling pathway in whole lung tissue, we cultured murine PCLS derived from TLR9 KO mice. The addition of mtDNA, from either PINK1 WT or KO mice, had no effect on total TGF-β levels indicating that TLR9 plays a key role in profibrotic signaling in response to mtDNA (Fig 5C). In summary, these data suggest that mtDNA oxidation state plays a role in its reactivity and mtDNA derived from PINK1 deficient cells has a greater potential to stimulate pro-fibrotic and pro-inflammatory responses.

### Circulating mitochondrial DNA levels correlate with severity of disease in IPF and is linked to PINK1 deficiency

**Fig 6:**
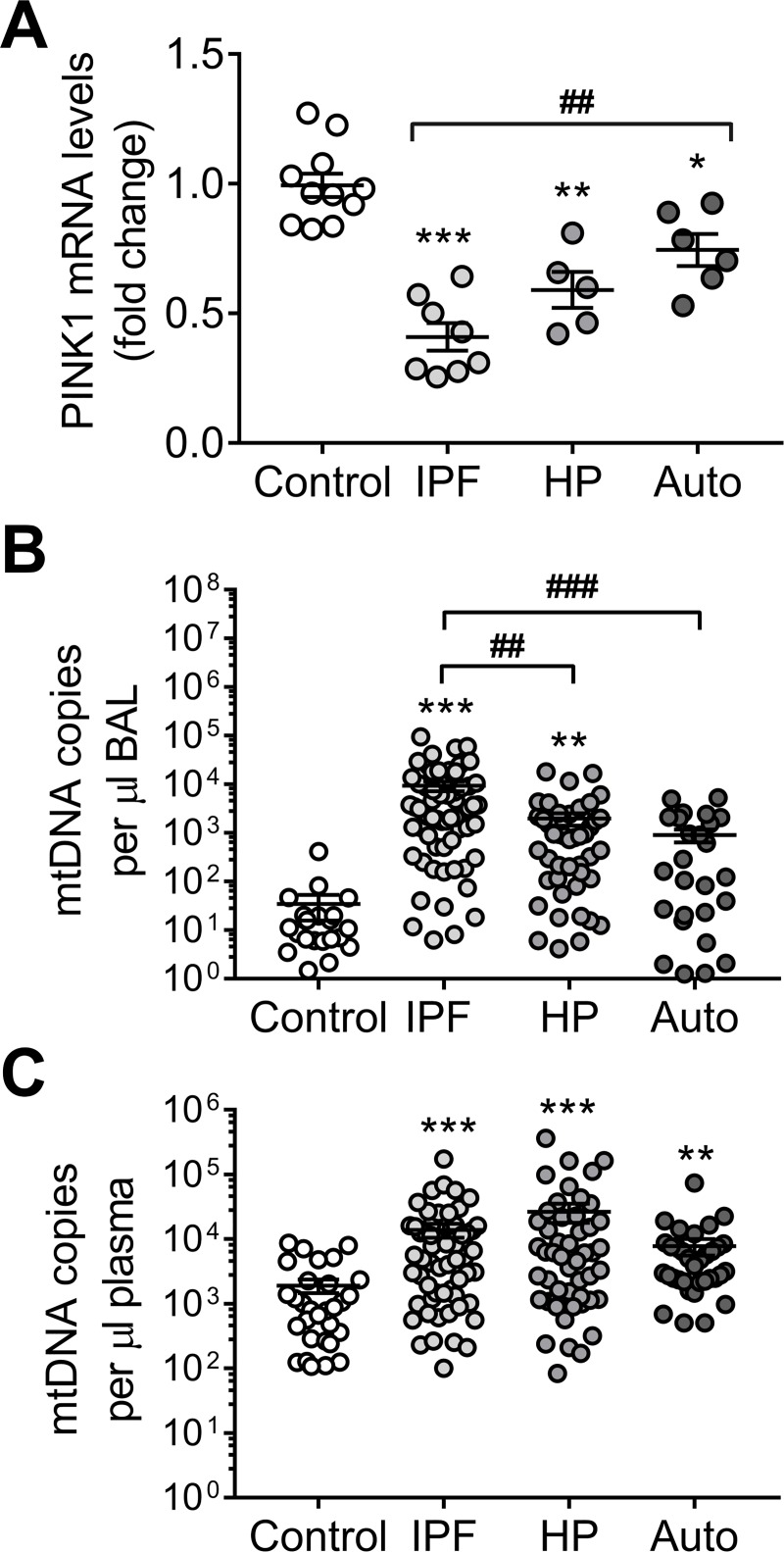
Mitochondrial DNA can be found in the bronchoalveolar lavage (BAL) and the plasma of patients with interstitial lung diseases (ILD). (A) PINK1 mRNA transcript levels in total lung tissue of aged-matched controls and samples from different ILD (IPF: idiopathic pulmonary fibrosis; HP: hypersensitivity pneumonitis; Auto: autoimmune-related ILD) (n = 10 control, n = 8 IPF, n = 5 HP, n = 6 Auto; *p<0.01, **p<0.001 and ***p<0.0001 vs Control, ^##^p<0.001; two-way ANOVA with multiple comparisons). See S4 Table for demographic details about this cohort. (B) Detected mtDNA copies by qPCR in the BAL of aged-matched controls and patients with different ILD (n = 22 control, n = 63 IPF, n = 47 HP, n = 29 Auto; **p<0.001 and ***p<0.0001 vs Control, ^##^p<0.01 and ^###^p<0.001; two-way ANOVA with multiple comparisons). (C) Detected mtDNA copies by qPCR in the plasma of aged-matched controls and patients with different ILD (n = 30 control, n = 60 IPF, n = 50 HP, n = 35 Auto; **p<0.001 and ***p<0.0001 vs Control; two-way ANOVA with multiple comparisons). Dot plots represent mean ± SEM. See S6B–S6D Fig for details regarding nuclear DNA detection. See S5 Table for demographic details of patients reported in panel B-C.

Cell-free mtDNA is shed into circulation in many medical conditions that involve significant tissue injury [15, 16, 28] and in IPF [18]. To investigate the link between PINK1 levels, cell-free mtDNA and ILD, we obtained plasma and bronchoalveolar lavage (BAL) samples from a cohort of patients from the Interstitial Lung Disease Clinic of the National Institute of Respiratory Diseases in Mexico City. This cohort included healthy controls and patients diagnosed with IPF, hypersensitivity pneumonitis (HP) or autoimmune-related ILD (S5 Table). First, we confirmed that lungs from IPF patients (which are characterized by high levels of TGFβ [29–31]) show low PINK1 expression, compared with age-matched donor controls; but also do other ILD, in a smaller magnitude (Fig 6A and S4 Table). In the patients, plasma and BAL fluids were obtained at the moment of diagnosis and before any treatment was indicated. In BAL, mtDNA was statistically increased in all the ILD disease groups compared with controls. Of note, BAL mtDNA levels were statistically higher in IPF when compared with HP and autoimmune-related ILD (Fig 6B and S7 Table). In plasma, while the level of circulating mtDNA was increased in all three groups compared to controls, no difference were found among them (Fig 6C and S8 Table). In IPF patients, increased levels of circulating mtDNA in plasma correlated with lower diffusing capacity for carbon monoxide (DLCO), lower percentage of forced expiratory volume for 1 second (FEV1%), and poorer performance on a 6-minute walk (6MWD) test supporting a direct correlation between levels of circulating mtDNA and IPF severity (S6A Fig). Although the release of mtDNA demonstrates potential clinical utility in IPF [18], these data suggest that it could be possible to expand those studies to other ILDs since all share a lower expression of PINK1 linked to mtDNA release.

### Mitochondrial DNA crosstalk signal between epithelial cells and fibroblasts

**Fig 7:**
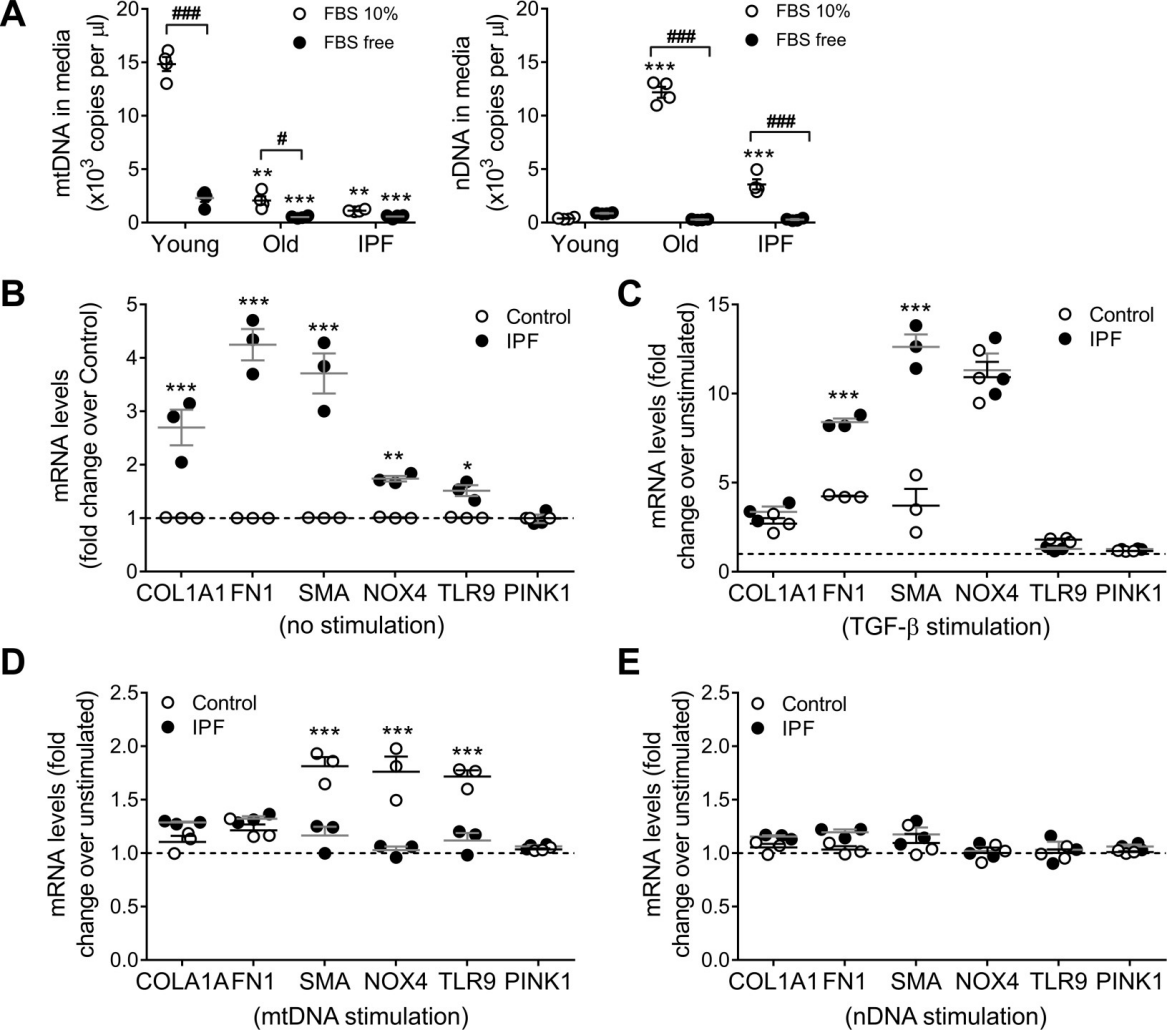
Lung epithelial cells derived mitochondrial DNA has an effect on lung fibroblast. (A) Mitochondrial DNA and nuclear release to the media by human lung fibroblast from young (33±4), old age-matched controls (67±3) or IPF patient (68±4) after 24h in culture with different concentrations of FBS (fetal bovine serum) added to the culture media (n = 4; ***p<0.001 and **p<0.01 vs Young, ^###^p<0.001; two-way ANOVA with multiple comparisons). (B) Baseline expression (non-stimulated) of different pro-fibrotic genes in age-matched human lung fibroblast from control and IPF patients (n = 3; ***p<0.001, **p<0.005, *p< 0.05 vs control; two-way ANOVA with multiple comparisons). (C) Expression of different pro-fibrotic genes in age-matched human lung fibroblast from control and IPF patients after 48h of TGF-β stimulation (5ng/ml) (n = 3; ***p<0.001, vs unstimulated; two-way ANOVA with multiple comparisons). (D) Fold change (over non-stimulated baseline) of pro-fibrotic markers expression after 48h of stimulation with 1μg/ml of exogenous mtDNA in age-matched human lung fibroblast from control and IPF patients (n = 3; ***p<0.001 vs unstimulated; two-way ANOVA with multiple comparisons). (E) Fold change (over non-stimulated baseline) of pro-fibrotic markers expression after 48h of stimulation with 1μg/ml of nuclear DNA in age-matched human lung fibroblast from control and IPF patients (n = 3; two-way ANOVA with multiple comparisons).

To test if the role of fibroblast as the source of mtDNA release, we isolated human lung primary fibroblast from young donors (33±4), old age-matched controls (67±3) or IPF patient (68±4). As we previously reported [4], there is no change in PINK1 expression between IPF and non-IPF lung isolated fibroblasts. After 24h of culture in FBS free media there was no differential release of mtDNA in to the supernatant from age-matched control or IPF fibroblast, only small contribution from the normal young fibroblast (Fig 7A). When the fibroblasts were cultured in 10% FBS, young donor derived fibroblast differentially release mtDNA but neither the old or IPF fibroblast since the mtDNA detected in their media correlate with the leak of nuclear DNA (probably due to cell death) (Fig 7A).

**Fig 8:**
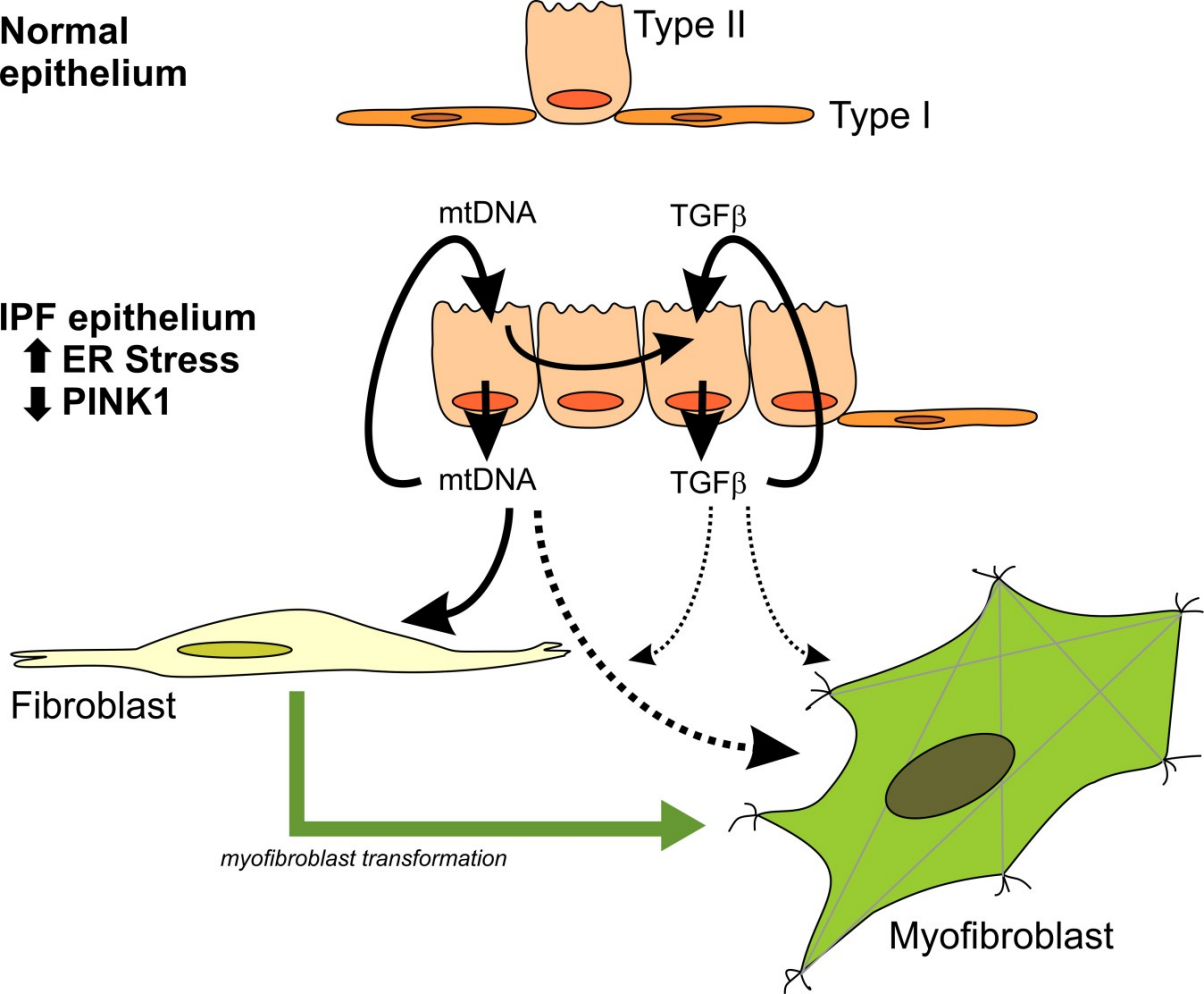
Mitochondrial DNA release has a key role in the progression of fibrosis. Chronically injured, ER-stress sensitive, PINK1-deficient AECII in IPF lungs are the driver to this pro-fibrotic signaling through the release of mtDNA and upregulation of TGFb (via a TLR9-NFκB axis) and it will affect the effector cell (the fibroblast) in different ways. TFGb release could perpetuate the migration of wound healing responding fibroblast to this microenvironment, while mtDNA DAMPs could be key to the progression of the disease by transforming the pool of these fibroblasts to a more pro-fibrotic myofibroblast-like phenotype.

Non-stimulated primary human lung fibroblasts isolated from IPF lungs had increased expression of pro-fibrotic markers (collagen and fibronectin), markers of activation (α-SMA and Nox4), and a slight higher expression of TLR9 (1.5-fold) compared to fibroblasts isolated from aged-matched non-IPF controls (IPF = 67±3 y; Control = 68±4 y) (Fig 7B). To demonstrate that both IPF and non-IPF fibroblasts were capable of increasing the expression of these markers of fibrosis they were treated with 5ng/mL TGF-β for 48 hours. Both groups of fibroblast upregulated the expression of collagen, Nox4, fibronectin and α-SMA; while TLR9 and PINK1 expression remained unchanged. However, the relative increase in fibronectin (IPF, 8-fold vs non-IPF, 4-fold) and α-SMA (IPF, 13-fold vs non-IPF, 3-fold) was significantly greater in IPF fibroblasts (Fig 7C). To test the reactivity of fibroblast to exogenous DNA, we treated IPF and non-IPF lung fibroblasts with 1 μg/mL of mtDNA or nDNA for 48 hours. Upon stimulation with mtDNA, only the non-IPF derived fibroblast show statistically significant increase in α-SMA, Nox4, and TLR9 (Fig 7D). Primary human lung fibroblasts exposed to nDNA did not show changes in the expression of the reposted marker (Fig 7E) regardless of their origin. These findings suggest that non-IPF fibroblasts have a pro-fibrotic response to mtDNA DAMPS, while IPF fibroblasts that already show a myofibroblast-like phenotype are less sensitive to this stimulus (Fig 8).

## Discussion

Our previous studies have shown that AECII of IPF patients have altered mitochondrial morphology and function associated with deficiency of PINK1 [4]. In this study, we report that low levels of PINK1 in AECII resulted in mitochondrial DNA damage and release of mtDNA. Released mtDNA was recognized by TLR9 inducing downstream TGF-β-mediated pro-fibrotic responses, as well as the well-known established inflammatory mediators for this pathway. This pro-fibrotic response was originated in AECII by a NFκB dependent mechanism and led to lung fibroblast activation. We have also shown that increased levels of circulating mtDNA are found in IPF as well as HP and ILD associated with autoimmune diseases patients. However, in bronchoalveolar fluids mtDNA was significantly increased in IPF compared to the other two ILDs, and moreover, a significant correlation between levels of plasma mtDNA and disease severity was found only in IPF patients.

Damaged mitochondria have been implicated in the induction of inflammation through the production of reactive oxygen species and the release of DAMPS, including mtDNA. For instance, deficiency of Parkin and PINK1 in mice under the stress of exhaustive exercise, provoke cytosolic release of mtDNA with activation of the STING-IRF3-dependent signaling pathway inducing interferon-mediated inflammatory responses [32]. Interestingly, our results revealed that leakage of mtDNA in the lung of PINK1 deficient mice can also induce inflammatory and fibrotic responses through the TLR9 endosomal pathway. A potential explanation is that PINK1 and Parkin not only regulate mitophagy but also participate in the modulation of mitochondrial endosomal degradation, a pathway where mitochondria can be sequestered inside the early endosomes and subsequently be delivered to lysosomes for degradation and clearance [33].

TLR9 recognition of mtDNA DAMPs leading to pro-fibrotic signaling through upregulation, activation, and release of TGF-β has been previously described in other fibrotic disorders such as systemic sclerosis and in a model of prostate cancer [34, 35]. The primary function of TLR9 is to recognize viral or bacterial DNA pathogen-associated molecular patterns, specifically hypomethylated CpG motifs, and initiate an innate immune response through upregulation of inflammatory mediators such as IL-6, IL-12, and TNF in response to the presence of these pathogens. TLR9 recognition of mtDNA is likely an unfortunate consequence of the theorized origins of mitochondria as incorporated prokaryotic symbionts. In lung tissue, TLR9 is generally expressed on professional immune cells such as resident B cells and dendritic cells, but is upregulated in both, lung epithelial cells and fibroblasts in various disease states [36, 37]. Interestingly, increased TLR9 expression has been reported in IPF lung from patients who rapidly deteriorated, and in this context, activation of TLR9 signaling has been implicated in accelerated progression of IPF [38]. In epithelial cells, transcription of TLR9 can be upregulated by activation of TLR9 signaling but in the presence of severe cell stress NF-kB signal can directly modify TLR9 transcription [39, 40]. In addition, stimulating TLR9 signaling with CpG-containing oligonucleotides in normal fibroblast is capable of inducing a more invasive phenotype on those cells [41, 42]. While those experiments were conducted with CPG ODN rather than mtDNA as a TLR9 agonist, our findings that show that mtDNA derived from PINK1-depleted AECII can be a more effective driver of fibroblast activation in the progression of IPF. Finally, previous studies using Balb/c TLR9 deficient mice have failed to demonstrate a role of TLR9 in the pathogenesis of lung fibrosis because this background is resistant to the classical model of bleomycin induced lung fibrosis [43]. Moreover, TLR9 deficient mice have attenuated antiviral responses (by a higher production of IFN-β) limiting its use in the MHV68-induced lung fibrosis model [43].

Previous studies have shown increase of circulating mtDNA in plasma and BAL of IPF patients and strong association with disease progression [18]. We found that high levels of circulating mtDNA are not exclusive of IPF (in the context of ILDs) but only in this disease, the levels correlate with the severity of the disease at the time of diagnosis. Whether the mechanisms of mtDNA release in other ILD is different from the one in IPF or if there is a threshold in the microenvironment were the effect of mitochondrial DAMPs is even more deleterious, will require further studies. In IPF, our data suggest that defective mitochondrial homeostasis by PINK1 deficiency in AECII leads to oxidative damage and release of mtDNA with the subsequent activation of TLR9 and expression of TGF-β. The AECII-derived mtDNA DAMPs could play a key role in the progression of the disease by transforming the pool of normal fibroblast to a more pro-fibrotic myofibroblast-like phenotype. Thus, potential therapeutic approaches that target the clearance of damage mitochondria might be and effective therapy for this complex disease.

## Supporting information

S1 Table Demographic characteristics of lung’s patient cohort in Fig 4D. (DOCX)

S2 Table Demographic characteristics of lung’s patient cohort in Fig 4E. (DOCX)

S3 Table Demographic characteristics of lung’s patient cohort in Fig 4F. (DOCX)

S4 Table Demographic characteristics of lung’s patient cohort in Fig 6A. (DOCX)

S5 Table Baseline characteristics of the Mexico City’s cohort. (DOCX)

S6 Table Probe assays used in this work. (DOCX)

S7 Table Statistical analysis of mtDNA in BAL for the Mexico City’s cohort. (A) The spearman correlation between BAL mtDNA levels with clinical variables by diagnosis. (B) The distribution of BAL mtDNA by diagnosis.
(DOCX)

S8 Table Statistical analysis of mtDNA in plasma for the Mexico City’s cohort. (A) The spearman correlation between plasma mtDNA levels with clinical variables by diagnosis. (B) The distribution of plasma mtDNA by diagnosis.
(DOCX)

S1 Fig Cells treated with tunicamycin express higher levels of ER stress markers. (A) Levels of mRNA of ER stress markers BiP and CHOP in A549 cells treated for 24 with 0.1mg/ml of tunicamycin. (n = 4; ***p<0.0001; two-way ANOVA with multiple comparison). (B) BiP and CHOP mRNA levels in primary human epithelial cells treated for 24 with 0.1μg/ml of tunicamycin. (n = 4; ***p<0.0001; two-way ANOVA with multiple comparison). (C) PINK1 mRNA PINK1 knock-down A549 cells (n = 4; ***p<0.0001; unpaired t-test). (D) PINK1 mRNA PINK1 +/+ and -/- total lung lysate (n = 4; ***p<0.0001; ND non detectable; unpaired t-test). (E) PINK1 in A549 cells overexpressing GFP or PINK1 (n = 4; ***p<0.0001; unpaired t-test). Dot plots represent mean ± SEM.
(TIF)

S2 Fig PINK1 deficiency and free mitochondrial DNA increase pro-inflammatory and pro-fibrotic markers. (A) Dose-dependent release of TGF-β to the supernatant in mouse lung epithelial cell line MLE12 when treated extracellular mtDNA for 24h. (n = 3, *p<0.01; one-way ANOVA with multiple comparison). (B) IL6 transcript levels in A549 after 24h of 1μg/ml mtDNA stimulation (n = 3, *p<0.05; unpaired t-test). (C) IL6 mRNA in total lung lysate and TGF-β in BAL from PINK1 deficient mice (n = 3–5, *p<0.001; unpaired t-test). (D) IL6 mRNA levels and TGF-b released in the cell media in A549 after PINK1 knock-down (n = 4, *p<0.005; unpaired t-test). Dot plots represent mean ± SEM.
(TIF)

S3 Fig TLR9 mediates IL6 upregulation in response to mitochondrial DNA. (A) TLR9 mRNA TLR9 knock-down A549 cells (n = 4; ***p<0.0001; unpaired t-test). (B) IL6 mRNA transcript levels in primary human lung epithelial cells after stimulations with extracellular mtDNA (1μg/ml) in the presence of DNAse (1U/ml), pre-treatments with TRL9 antagonist ODN-TAG (1μM) or exposed to 1μg/ml of nDNA after 24h. TRL9 agonist ODN M362 (1μM) (n = 4, *p<0.01 vs PBS, #p<0.01 vs mtDNA; two-way ANOVA with multiple comparison). Dot plots represent mean ± SEM.
(TIF)

S4 Fig PINK1 deficient lungs show higher mitochondrial DNA oxidation. (A) Oxidative damage by 8-OH-dG in mtDNA, from total lung isolated mitochondria, in aged mice and PINK1 deficient mice -/- (n = 3; *p<0.01 vs 3 months old, #p<0.01 vs PINK1 +/+; two-way ANOVA with multiple comparison). (B) PINK1 levels in PINK1 knock-down A549 cells. (n = 4; *p<0.01; unpaired t-test). (C) H_2_O_2_-induced mtDNA lesions in PINK1 knock-down A549 cells at time of injury (400 μM H2O2 for 15 minutes) and after wash out (1h wash in new media) (n = 3; *p<0.01; two-way ANOVA with multiple comparison). Dot plots represent mean ± SEM.
(TIF)

S5 Fig Precision-cut lung tissue slices viability data. (A) Representative viability staining for human PCLS at time of process showing healthy cells in green. (B) Representative viability staining and quantification for precision-cut lung tissue slices at time of process (C57BL6 and TRL9 KO). Min-to-max box and whiskers.
(TIF)

S6 Fig Mitochondrial DNA in plasma of IPF patients correlate with marker of disease severity. (A) IPF circulating levels of mtDNA in plasma correlates with functional characteristics and markers of disease severity forced expiratory volume for 1 second (FEV1% n = 43), diffusing capacity for carbon monoxide DLCO% (n = 32) and distance on a 6-minute walk test (6MWD n = 25). (B) Levels of nuclear DNA (gDNA) detected in plasma of IPF patients does not correlate with the mtDNA copy numbers found (n = 60). Also, copy numbers of mtDNA in BAL does not correlate with mtDNA copy numbers in plasma (n = 12). (C) IPF circulating levels of nuclear DNA in plasma does not correlate with functional characteristics and markers of disease severity FEV1% (n = 43), DLCO% (n = 32) or 6MWD (n = 25). (D) Hypersensitivity pneumonitis (HP) circulating levels of mtDNA in plasma does not significantly correlate with functional characteristics and markers of disease severity FEV1% (n = 37), DLCO% (n = 22) or 6MWD (n = 4). Spearman correlation with graphical representation of the 95% confidence range.
(TIF)
