# Supplementary material for: PINK1 attenuates mtDNA release in alveolar epithelial cells and TLR9 mediated profibrotic responses
Source: PLoS One. 2019 Jun 6;14(6):e0218003. doi: 10.1371/journal.pone.0218003 (PMC6553779; doi:10.1371/journal.pone.0218003)
Supplement: S2 Table — (DOCX) [file pone.0218003.s002.docx]

**S2 Table. Demographic characteristics of lung’s patient cohort in Fig 4E**

|  | **Donor-Control**  **Young** | **Donor-Control**  **Old** | **IPF** |
| --- | --- | --- | --- |
| **Subjects** | 10 | 10 | 10 |
| **Age** | 35±6  (24 – 48) | 75±4  (62 – 79) | 69±4  (64 – 74) |
| **Gender** |  |  |  |
| **Female** | 4 (40%) | 4 (40%) | 5 (50%) |
| **Male** | 6 (60%) | 6 (60%) | 5 (50%) |
