# Supplementary material for: PINK1 attenuates mtDNA release in alveolar epithelial cells and TLR9 mediated profibrotic responses
Source: PLoS One. 2019 Jun 6;14(6):e0218003. doi: 10.1371/journal.pone.0218003 (PMC6553779; doi:10.1371/journal.pone.0218003)
Supplement: S3 Table — (DOCX) [file pone.0218003.s003.docx]

**S3 Table. Demographic characteristics of lung’s patient cohort in Fig 4F**

|  | **Donor-Control**  **Young** | **Donor-Control**  **Old** | **IPF** |
| --- | --- | --- | --- |
| **Subjects** | 6 | 6 | 6 |
| **Age** | 35±6  (25 – 43) | 65±9  (56 – 80) | 63±7  (54 – 70) |
| **Gender** |  |  |  |
| **Female** | 3 (50%) | 3 (50%) | 2 (34%) |
| **Male** | 3 (50%) | 3 (50%) | 3 (66%) |
