# Supplementary material for: PINK1 attenuates mtDNA release in alveolar epithelial cells and TLR9 mediated profibrotic responses
Source: PLoS One. 2019 Jun 6;14(6):e0218003. doi: 10.1371/journal.pone.0218003 (PMC6553779; doi:10.1371/journal.pone.0218003)
Supplement: S4 Table — (DOCX) [file pone.0218003.s004.docx]

**S4 Table. Demographic characteristics of lung’s patient cohort in Fig 6A**

|  | **Control** | **IPF** | **HP** | **Auto** |
| --- | --- | --- | --- | --- |
| **Subjects** | 10 | 8 | 5 | 6 |
| **Age** | 63±7  (55 – 70) | 67±3  (62 – 73) | 66±7  (58 – 76) | 60±6  (54 – 67) |
| **Gender** |  |  |  |  |
| **Female** | 4 (40%) | 4 (50%) | 2 (40%) | 3 (50%) |
| **Male** | 6 (60%) | 4 (50%) | 3 (60%) | 3 (50%) |
