# Supplementary material for: PINK1 attenuates mtDNA release in alveolar epithelial cells and TLR9 mediated profibrotic responses
Source: PLoS One. 2019 Jun 6;14(6):e0218003. doi: 10.1371/journal.pone.0218003 (PMC6553779; doi:10.1371/journal.pone.0218003)
Supplement: S5 Table — (DOCX) [file pone.0218003.s005.docx]

**S5 Table. Baseline characteristics of the Mexico City cohort**

|  | **Control** | **IPF** | **HP** | **Autoimmune** |
| --- | --- | --- | --- | --- |
| **Subjects** | 29 | 109 | 95 | 58 |
| **Age** |  |  |  |  |
| **Years (mean ± SD)** | 45±18 | 65±9 | 52±10 | 58±10 |
| **Rage** | (18 – 77) | (42 – 86) | (21 – 74) | (35 – 76) |
| **Sex, n (%)** |  |  |  |  |
| **Female** | 10 (34%) | 25 (23%) | 76 (80%) | 37 (64%) |
| **Male** | 19 (66%) | 84 (77%) | 19 (20%) | 21 (36%) |
| **Smoking status, n (%)** |  |  |  |  |
| **Never** | 9 (31%) | 23 (21%) | 34 (36%) | 26 (45%) |
| **Former** | 1 (3%) | 37 (34%) | 12 (13%) | 11 (19%) |
| **Unknown** | 19 (66%) | 49 (45%) | 49 (51%) | 21 (36%) |
| **FVC % (mean ± SD)** | 96±14 | 70±22 | 62±23 | 65±22 |
| **DLCO % (mean ± SD)** | 132±15 | 55±25 | 49±25 | 50±31 |
| Definition of abbreviations. IPF: idiopathic pulmonary fibrosis; HP: hypersensitivity pneumonitis; Autoimmune: autoimmune-related ILD; FVC: forced vital capacity, DLCO: diffusing capacity for carbon monoxide | | | | |
