# Supplementary material for: PINK1 attenuates mtDNA release in alveolar epithelial cells and TLR9 mediated profibrotic responses
Source: PLoS One. 2019 Jun 6;14(6):e0218003. doi: 10.1371/journal.pone.0218003 (PMC6553779; doi:10.1371/journal.pone.0218003)
Supplement: S6 Table — (DOCX) [file pone.0218003.s006.docx]

**S6 Table. Probe assays used in this work**

| **Species** | **Gene** | **Probe Assay** |
| --- | --- | --- |
| Human | RN18S | Hs.PT.47.122532.g |
| Mouse | RN18S | Mm.PT.45.122532.g |
| Human | PINK1 | Hs.PT.56a.27157402 |
| Mouse | PINK1 | Mm.PT.56a.23711353 |
| Human | IL6 | Hs.PT.56a.40226675 |
| Mouse | IL6 | Mm.PT.49a.11799101.g |
| Human | TGFB1 | Hs.PT.56a.39813975 |
| Mouse | TGFB1 | Mm.PT.47.12668954 |
| Human | IL1B | Hs.PT.56a. 1518186 |
| Mouse | IL1B | Mm.PT.49a.17212823 |
| Human | TLR9 | Hs.PT.56a.40576968 |
| Mouse | TLR9 | Ms.PT.56a.5114450 |
| Human | COL1A1 | Hs.PT.56a.15517795 |
| Human | FN1 | Hs.PT.58.40005963 |
| Human | NOX4 | Hs.PT.47.1114634 |
| Human | ACTA2 | Hs.PT.56a.2542642 |
