# Supplementary material for: PINK1 attenuates mtDNA release in alveolar epithelial cells and TLR9 mediated profibrotic responses
Source: PLoS One. 2019 Jun 6;14(6):e0218003. doi: 10.1371/journal.pone.0218003 (PMC6553779; doi:10.1371/journal.pone.0218003)
Supplement: S7 Table — (A) The spearman correlation between BAL mtDNA levels with clinical variables by diagnosis. (B) The distribution of BAL mtDNA by diagnosis. (DOCX) [file pone.0218003.s007.docx]

**S7 Table A. The spearman correlation between BAL mtDNA levels with clinical variables by diagnosis.**

|  | **IPF** | | | **HP** | | | **Autoimmune** | | |
| --- | --- | --- | --- | --- | --- | --- | --- | --- | --- |
|  | **n** | **r** | **p** | **n** | **r** | **p** | **n** | **r** | **p** |
| **Age** | 61 | 0.16 | 0.2 | 58 | 0.35 | 0.008 | 28 | -0.12 | 0.5 |
| **FVC %** | 61 | -0.05 | 0.7 | 57 | 0.06 | 0.7 | 27 | -0.27 | 0.18 |
| **FEV1 %** | 61 | 0.02 | 0.9 | 57 | -0.13 | 0.3 | 27 | -0.31 | 0.12 |
| **FEV1/FVC** | 56 | 0.24 | 0.08 | 57 | -0.05 | 0.7 | 27 | -0.18 | 0.4 |
| **TLC** | 45 | -0.04 | 0.8 | 48 | -0.22 | 0.13 | 19 | -0.30 | 0.2 |
| **DLCO** | 48 | 0.14 | 0.3 | 43 | -0.24 | 0.12 | 19 | -0.15 | 0.5 |
| **Sat at rest** | 51 | 0.16 | 0.3 | 52 | -0.18 | 0.20 | 21 | -0.31 | 0.17 |
| **Sat at exc.** | 34 | 0.15 | 0.4 | 35 | -0.29 | 0.10 | 10 | -0.62 | 0.06 |
| **Meter** | 30 | 0.28 | 0.14 | 17 | -0.17 | 0.5 | 10 | -0.11 | 0.8 |
| *Definition of abbreviations*. IPF: idiopathic pulmonary fibrosis; HP: hypersensitivity pneumonitis; Autoimmune: autoimmune-related ILD; FVC: forced vital capacity; FEV1: forced expiratory volume during the first second; TLC: total lung capacity; DLCO: diffusing capacity for carbon monoxide; Sat: saturation | | | | | | | | | |

**S7 Table B. The distribution of BAL mtDNA by diagnosis**

| ***The distribution of BAL mtDNA by diagnosis.*** | | | | | |
| --- | --- | --- | --- | --- | --- |
|  | IPF  n=61 | HP  n=58 | Autoimmune  n=28 | Control  n=19 | Overall *p* value |
| mtDNA, median (interquartile range) | 807.6  (150.3-4835.0) | 455.6  (42.9-2420.0) | 218.5  (5.0-2960.8) | 55.7  (8.5-126.0) | 0.0002 |
| Post hoc *p* value from IPF vs. HP = 0.052  Post hoc *p* value from IPF vs. Autoimmune = 0.023  Post hoc *p* value from IPF vs. Control <0.001  Post hoc *p* value from HP vs. Control <0.001  Post hoc *p* value from Autoimmune vs. Control = 0.011 | | | | | |
