# Supplementary material for: PINK1 attenuates mtDNA release in alveolar epithelial cells and TLR9 mediated profibrotic responses
Source: PLoS One. 2019 Jun 6;14(6):e0218003. doi: 10.1371/journal.pone.0218003 (PMC6553779; doi:10.1371/journal.pone.0218003)
Supplement: S8 Table — (A) The spearman correlation between plasma mtDNA levels with clinical variables by diagnosis. (B) The distribution of plasma mtDNA by diagnosis. (DOCX) [file pone.0218003.s008.docx]

**S8 Table A. The spearman correlation between plasma mtDNA levels with clinical variables by diagnosis.**

|  | **IPF** | | | **HP** | | | **Autoimmune** | | | **Control** | | |
| --- | --- | --- | --- | --- | --- | --- | --- | --- | --- | --- | --- | --- |
|  | **n** | **r** | **p** | **n** | **r** | **p** | **n** | **r** | **p** | **n** | **r** | **p** |
| **Age** | 47 | -0.30 | 0.040 | 37 | -0.26 | 0.11 | 30 | -0.11 | 0.6 | 10 | 0.22 | 0.5 |
| **FVC %** | 43 | -0.28 | 0.06 | 37 | -0.26 | 0.12 | 29 | -0.13 | 0.5 | 10 | -0.39 | 0.3 |
| **FEV1 %** | 43 | -0.32 | 0.034 | 37 | -0.24 | 0.16 | 29 | -0.15 | 0.4 | 10 | -0.46 | 0.19 |
| **FEV1/FVC** | 22 | 0.02 | 0.9 | 37 | 0.30 | 0.08 | 29 | 0.12 | 0.5 | 10 | 0.55 | 0.10 |
| **TLC** | 30 | 0.01 | 0.9 | 29 | -0.02 | 0.9 | 21 | -0.07 | 0.8 | 10 | 0.04 | 0.9 |
| **DLCO** | 32 | -0.39 | 0.028 | 22 | -0.15 | 0.5 | 22 | -0.12 | 0.6 | 10 | -0.34 | 0.3 |
| **Sat at rest** | 40 | -0.25 | 0.12 | 31 | -0.28 | 0.13 | 24 | -0.19 | 0.4 | 10 | -0.57 | 0.09 |
| **Sat at exc.** | 28 | -0.47 | 0.012 | 14 | -0.16 | 0.6 | 10 | 0.26 | 0.5 | 10 | -0.66 | 0.038 |
| **Meter** | 25 | -0.47 | 0.018 | 4 | -0.40 | 0.6 | 9 | -0.03 | 0.9 | 10 | 0.18 | 0.6 |
| *Definition of abbreviations*. IPF: idiopathic pulmonary fibrosis; HP: hypersensitivity pneumonitis; Autoimmune: autoimmune-related ILD; FVC: forced vital capacity; FEV1: forced expiratory volume during the first second; TLC: total lung capacity; DLCO: diffusing capacity for carbon monoxide; Sat: saturation | | | | | | | | | | | | |

**S8 Table B. The distribution of plasma mtDNA by diagnosis**

| ***The distribution of plasma mtDNA by diagnosis.*** | | | | | |
| --- | --- | --- | --- | --- | --- |
|  | IPF  n=47 | HP  n=37 | Autoimmune  n=30 | Control  n=10 | Overall *p* value |
| mtDNA, median (interquartile range) | 3712.11  (667.33-13537.90) | 61688.91  (1292.59-19003.47) | 3070.07  (2248.86-7510.36) | 1851.8  (124.28-8769.4) | 0.0005 |
| Post hoc *p* value from IPF vs. Autoimmune = 0.4  Post hoc *p* value from IPF vs. Control <0.001  Post hoc *p* value from Autoimmune vs. Control <0.001  Post hoc *p* value from HP vs. Control <0.001  Post hoc *p* value from HP vs. IPF = 0.08 | | | | | |
