# Supplementary figures and images for: PINK1 attenuates mtDNA release in alveolar epithelial cells and TLR9 mediated profibrotic responses
Source: PLoS One. 2019 Jun 6;14(6):e0218003. doi: 10.1371/journal.pone.0218003 (PMC6553779; doi:10.1371/journal.pone.0218003)

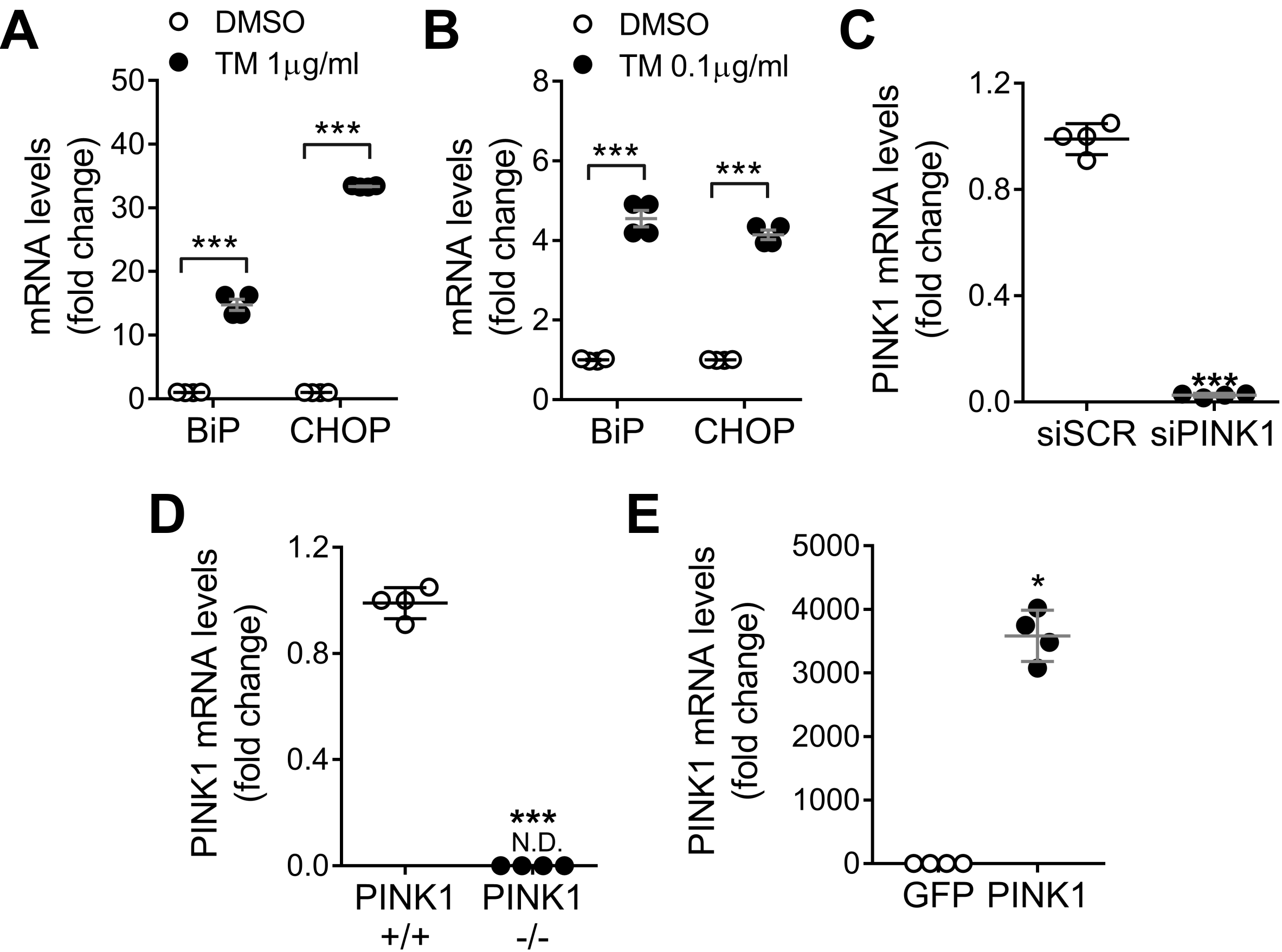

Supplement: S1 Fig — (A) Levels of mRNA of ER stress markers BiP and CHOP in A549 cells treated for 24 with 0.1mg/ml of tunicamycin. (n = 4; ***p<0.0001; two-way ANOVA with multiple comparison). (B) BiP and CHOP mRNA levels in primary human epithelial cells treated for 24 with 0.1μg/ml of tunicamycin. (n = 4; ***p<0.0001; two-way ANOVA with multiple comparison). (C) PINK1 mRNA PINK1 knock-down A549 cells (n = 4; ***p<0.0001; unpaired t-test). (D) PINK1 mRNA PINK1 +/+ and -/- total lung lysate (n = 4; ***p<0.0001; ND non detectable; unpaired t-test). (E) PINK1 in A549 cells overexpressing GFP or PINK1 (n = 4; ***p<0.0001; unpaired t-test). Dot plots represent mean ± SEM. (TIF) [file pone.0218003.s009.tif]

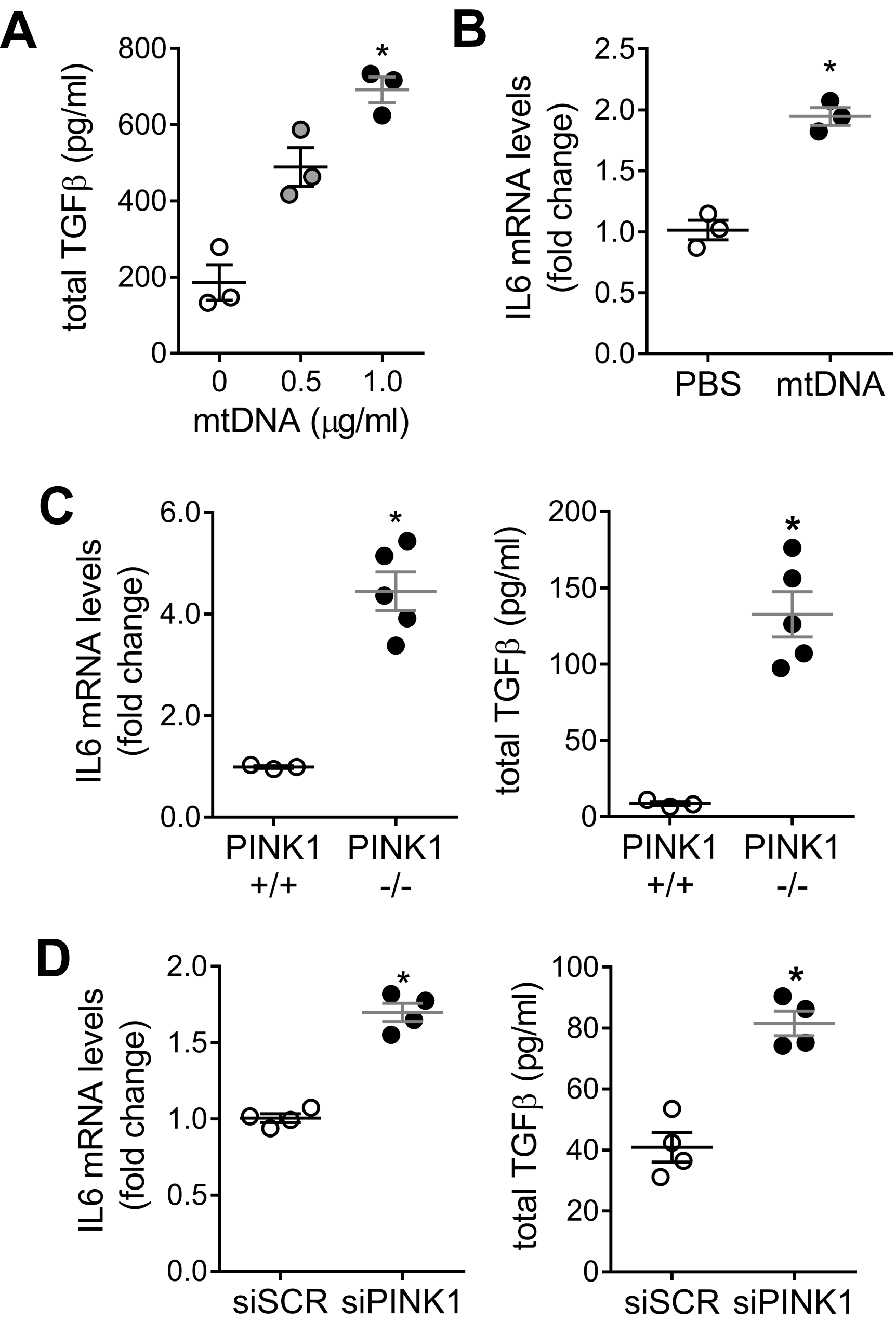

Supplement: S2 Fig — (A) Dose-dependent release of TGF-β to the supernatant in mouse lung epithelial cell line MLE12 when treated extracellular mtDNA for 24h. (n = 3, *p<0.01; one-way ANOVA with multiple comparison). (B) IL6 transcript levels in A549 after 24h of 1μg/ml mtDNA stimulation (n = 3, *p<0.05; unpaired t-test). (C) IL6 mRNA in total lung lysate and TGF-β in BAL from PINK1 deficient mice (n = 3–5, *p<0.001; unpaired t-test). (D) IL6 mRNA levels and TGF-b released in the cell media in A549 after PINK1 knock-down (n = 4, *p<0.005; unpaired t-test). Dot plots represent mean ± SEM. (TIF) [file pone.0218003.s010.tif]

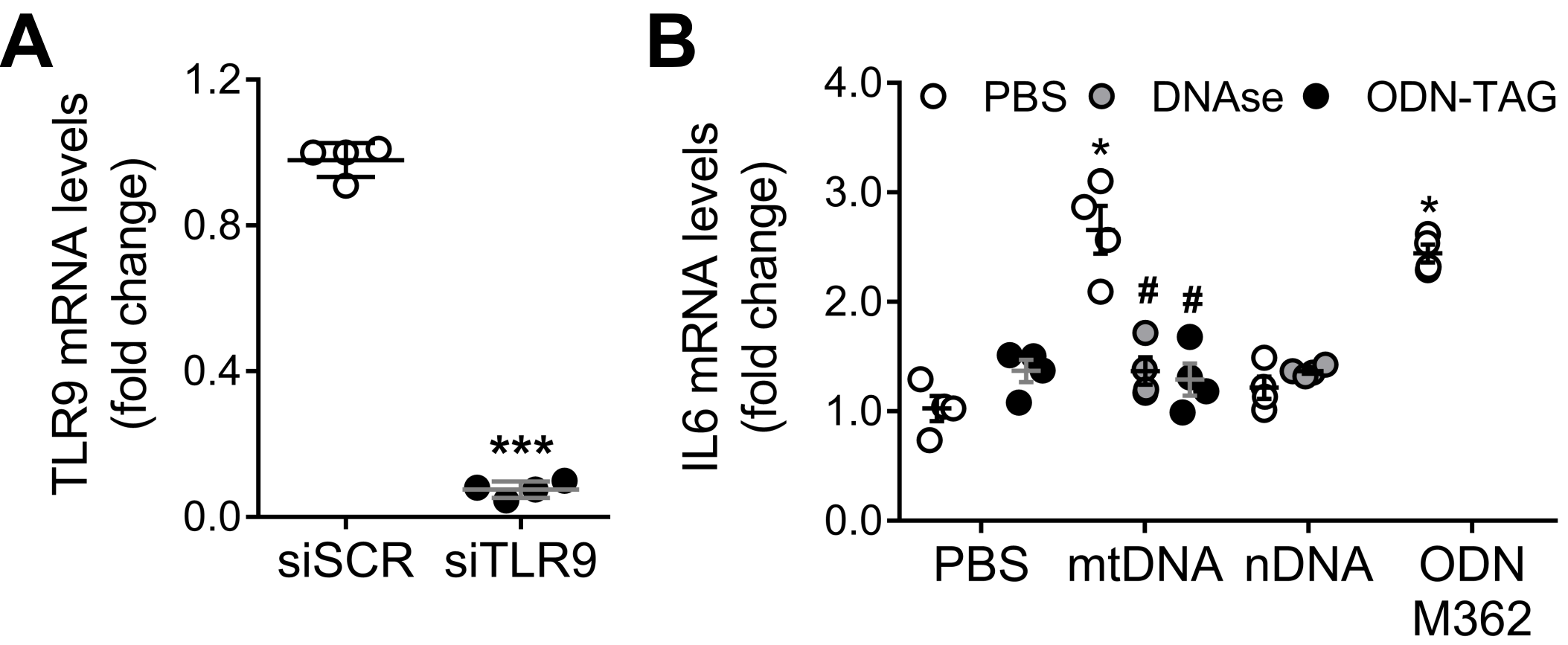

Supplement: S3 Fig — (A) TLR9 mRNA TLR9 knock-down A549 cells (n = 4; ***p<0.0001; unpaired t-test). (B) IL6 mRNA transcript levels in primary human lung epithelial cells after stimulations with extracellular mtDNA (1μg/ml) in the presence of DNAse (1U/ml), pre-treatments with TRL9 antagonist ODN-TAG (1μM) or exposed to 1μg/ml of nDNA after 24h. TRL9 agonist ODN M362 (1μM) (n = 4, *p<0.01 vs PBS, #p<0.01 vs mtDNA; two-way ANOVA with multiple comparison). Dot plots represent mean ± SEM. (TIF) [file pone.0218003.s011.tif]

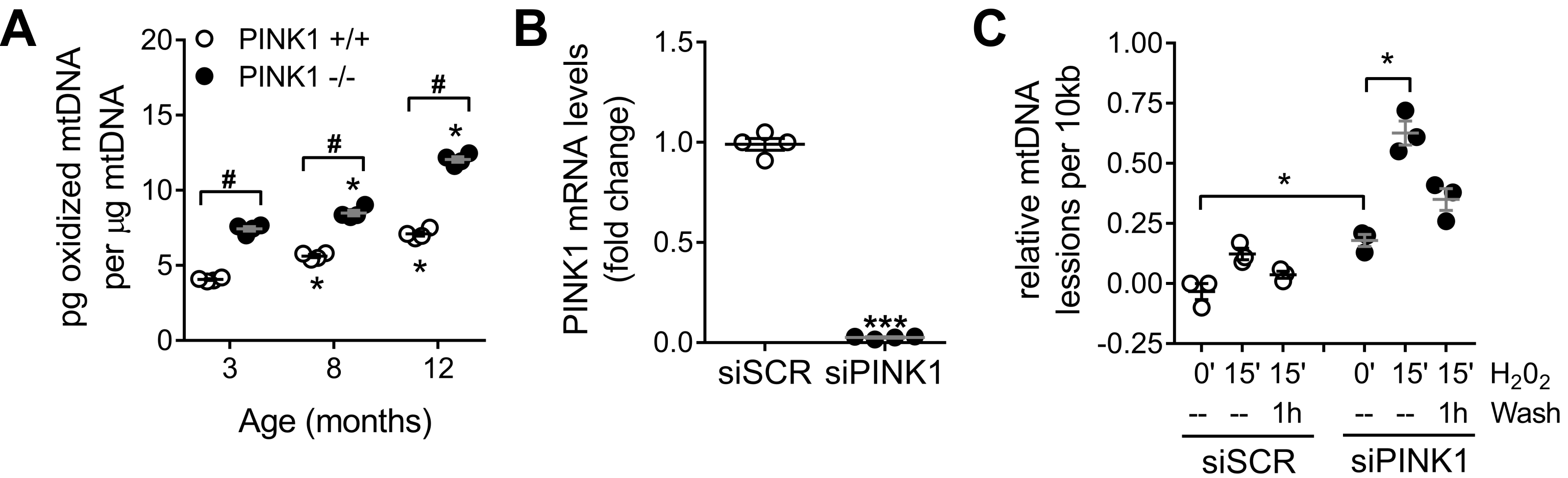

Supplement: S4 Fig — (A) Oxidative damage by 8-OH-dG in mtDNA, from total lung isolated mitochondria, in aged mice and PINK1 deficient mice -/- (n = 3; *p<0.01 vs 3 months old, #p<0.01 vs PINK1 +/+; two-way ANOVA with multiple comparison). (B) PINK1 levels in PINK1 knock-down A549 cells. (n = 4; *p<0.01; unpaired t-test). (C) H2O2-induced mtDNA lesions in PINK1 knock-down A549 cells at time of injury (400 μM H2O2 for 15 minutes) and after wash out (1h wash in new media) (n = 3; *p<0.01; two-way ANOVA with multiple comparison). Dot plots represent mean ± SEM. (TIF) [file pone.0218003.s012.tif]

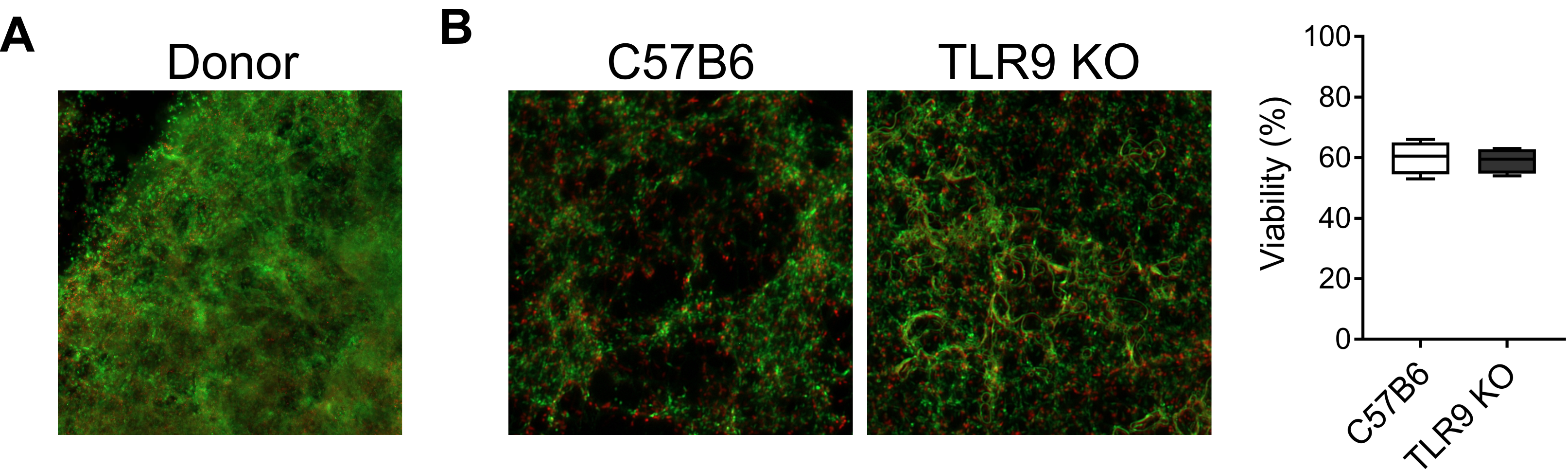

Supplement: S5 Fig — (A) Representative viability staining for human PCLS at time of process showing healthy cells in green. (B) Representative viability staining and quantification for precision-cut lung tissue slices at time of process (C57BL6 and TRL9 KO). Min-to-max box and whiskers. (TIF) [file pone.0218003.s013.tif]

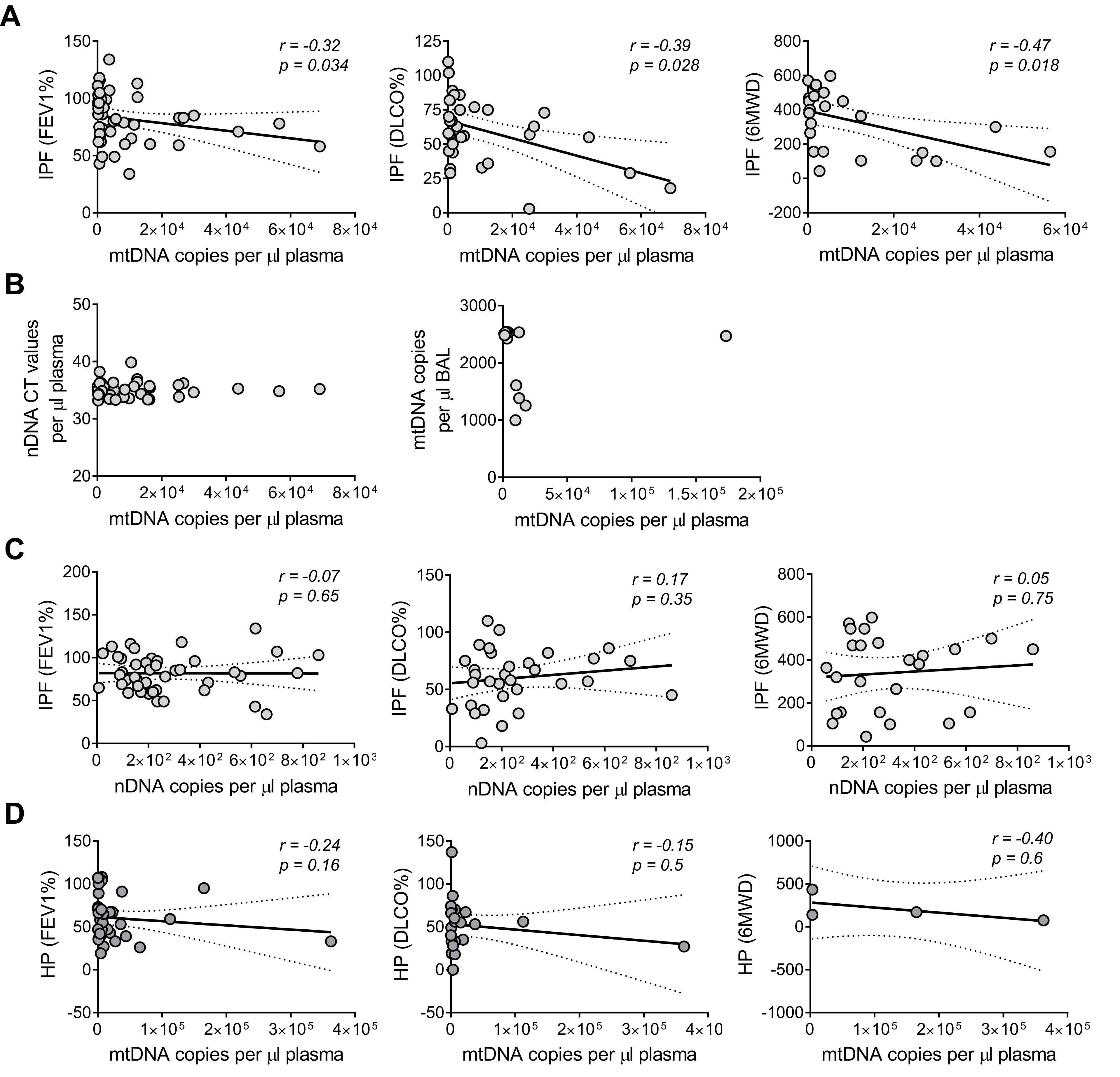

Supplement: S6 Fig — (A) IPF circulating levels of mtDNA in plasma correlates with functional characteristics and markers of disease severity forced expiratory volume for 1 second (FEV1% n = 43), diffusing capacity for carbon monoxide DLCO% (n = 32) and distance on a 6-minute walk test (6MWD n = 25). (B) Levels of nuclear DNA (gDNA) detected in plasma of IPF patients does not correlate with the mtDNA copy numbers found (n = 60). Also, copy numbers of mtDNA in BAL does not correlate with mtDNA copy numbers in plasma (n = 12). (C) IPF circulating levels of nuclear DNA in plasma does not correlate with functional characteristics and markers of disease severity FEV1% (n = 43), DLCO% (n = 32) or 6MWD (n = 25). (D) Hypersensitivity pneumonitis (HP) circulating levels of mtDNA in plasma does not significantly correlate with functional characteristics and markers of disease severity FEV1% (n = 37), DLCO% (n = 22) or 6MWD (n = 4). Spearman correlation with graphical representation of the 95% confidence range. (TIF) [file pone.0218003.s014.tif]
